# Persistent Exposure to *Porphyromonas gingivalis* Promotes Proliferative and Invasion Capabilities, and Tumorigenic Properties of Human Immortalized Oral Epithelial Cells

**DOI:** 10.3389/fcimb.2017.00057

**Published:** 2017-02-24

**Authors:** Fengxue Geng, Junchao Liu, Yan Guo, Chen Li, Hongyang Wang, Hongyan Wang, Haijiao Zhao, Yaping Pan

**Affiliations:** ^1^Department of Periodontics, School of Stomatology, China Medical UniversityShenyang, China; ^2^Key laboratory of Liaoning Province Oral Disease, School of Stomatology, China Medical UniversityShenyang, China; ^3^Department of Oral Biology, School of Stomatology, China Medical UniversityShenyang, China; ^4^Department of Medicine, the Center for Immunity, Inflammation & Regenerative Medicine, University of VirginiaCharlottesville, VA, USA

**Keywords:** *Porphyromonas gingivalis*, OSCC, long-term infection, inflammatory microenvironment, tumorigenic properties

## Abstract

Recent epidemiological studies revealed a significant association between oral squamous cell carcinoma (OSCC) and *Porphyromonas gingivalis*, a major pathogen of periodontal disease. As a keystone pathogen of periodontitis, *P. gingivalis* is known not only to damage local periodontal tissues, but also to evade the host immune system and eventually affect systemic health. However, its role in OSCC has yet to be defined. To explore the underlying effect of chronic *P. gingivalis* infection on OSCC and to identify relevant biomarkers as promising targets for therapy and prevention, we established a novel model by exposing human immortalized oral epithelial cells (HIOECs) to *P. gingivalis* at a low multiplicity of infection (MOI) for 5–23 weeks. The *P. gingivalis* infected HIOECs were monitored for tumor biological alteration by proliferation, wound healing, transwell invasion, and gelatin zymography assays. Microarray and proteomic analyses were performed on HIOECs infected with *P. gingivalis* for 15 weeks, and some selected data were validated by quantitative real-time PCR and (or) western blot on cells infected for 15 and 23 weeks. Persistent exposure to *P. gingivalis* caused cell morphological changes, increased proliferation ability with higher S phase fraction in the cell cycle, and promoted cell migratory and invasive properties. In combining results of bioinformatics analyses and validation assays, tumor-related genes such as NNMT, FLI1, GAS6, lncRNA CCAT1, PDCD1LG2, and CD274 may be considered as the key regulators in tumor-like transformation in response to long-time exposure of *P. gingivalis*. In addition, some useful clinical biomarkers and novel proteins were also presented. In conclusion, *P. gingivalis* could promote tumorigenic properties of HIOECs, indicating that chronic *P. gingivalis* infection may be considered as a potential risk factor for oral cancer. The key regulators detected from the present model might be used in monitoring the development of OSCC with chronic periodontal infection.

## Introduction

Worldwide, oral cancer accounts for 2–4% of all cancer cases. Oral squamous cell carcinoma (OSCC) is a common malignant epithelial neoplasm that constitutes 90% of all oral neoplasms. In addition, the incidence of OSCC has increased in recent years. The percentage of patients with 5-year survival from OSCC varies from 40 to 50%. As OSCC usually progresses without causing obvious pain or symptoms, patients usually ignore it in the early stages when definitive treatment may be provided locally within the oral cavity. Thus, to find useful diagnostic and therapeutic targets with easy access in the oral cavity may facilitate prompt recognition of OSCC (Severino et al., 2015).

The relationship between tumor and chronic inflammation was presented over a century ago (Grivennikov et al., 2010). Certain interactions between pathogens and host cells may induce an imbalance of immune-inflammation response, which may increase the mutation rate of normal cells and trigger a series of molecular events participating in mediation of host cells proliferation and malignant transformation (Huang et al., 2011; Guven Maiorov et al., 2013; Tribble et al., 2013). Recently, specific microbial infections are considered as casual factors of cancer. The role of *Porphyromonas gingivalis* in OSCC has been investigated.

Periodontitis is a public health problem commonly suffered by adults worldwide (Van Dyke et al., 2015). *P. gingivalis*, the major pathogen of chronic periodontitis, produces various virulence factors that may affect host immune response, modify host gene expression, and degrade host cell proteins or surface receptors (Yilmaz, 2008). Colonization of *P. gingivalis* is not only limited to periodontal tissues, but spreads in initial lesion sites of OSCC such as the buccal and tongue mucosa (Atanasova and Yilmaz, 2015). A recent meta-analysis indicated that the presence of *P. gingivalis* increased the chance of cancer development and periodontal disease as much as 1.36 times [odds ratio (OR), 1.36; 95% confidence interval (CI), 0.47–3.97; Sayehmiri et al., 2015]. Specific to OSCC, the number of oral bacteria isolated at ulcerating surfaces of OSCC tissues was significantly higher than that at normal mucosa, while the genus Porphyromonas showed the highest rates of isolation (Nagy et al., 1998). More recently, the presence of *P. gingivalis* in gingival carcinoma tissues was reported to be more than 33% higher than that in normal gingival tissues, while the intensity of *P. gingivalis* staining was also significantly enhanced in malignant tissues compared with other non-invasive bacteria such as *Streptococcus gordonii* (Katz et al., 2011). Our group also found that the prevalence ratio of *P. gingivalis* in OSCC tissues was higher than that in normal tissues. Interestingly, in malignant tissues, *P. gingivalis* gathered around cell nuclei with obvious heterogeneity (data not yet published). However, it was undefined whether *P. gingivalis* indeed played a stimulating role in the early stages of OSCC or only invaded into the transformed malignant cells.

Cancer is manifested as a proliferation of host cells without control (Plottel and Blaser, 2011). As reported, *P. gingivalis* could promote growth of primary gingival epithelial cells (GECs) after infection for 24 h at a multiplicity of infection (MOI) of 100 or 10 (Kuboniwa et al., 2008). Similarly, our previous study showed that *P. gingivalis* could promote proliferation of immortalized human gingival epithelial (IHGE) cells by accelerating cell cycle progression between 10 and 12 h at an MOI of 100 (Pan et al., 2014). *P. gingivalis* could also increase proliferation of primary periodontal ligament fibroblasts (PDLFs) with G1 phase promotion at 6 h with an MOI of 100 (Liu et al., 2015). In addition, in GECs, infection by *P. gingivalis* in the early stage can regulate the production of reactive oxygen species (ROS; Choi et al., 2013), the key factors inducing DNA damage and genomic instability within an inflammatory microenvironment (Grivennikov et al., 2010). During short-term infection, *P. gingivalis* can also modulate the expression of some key factors which mediate cancer development and progression (Yilmaz et al., 2004; Groeger et al., 2011; Inaba et al., 2014; Sztukowska et al., 2015; Zhou et al., 2015). Hence, we hypothesized that chronic infection by *P. gingivalis* might play a promoting role in tumor-like transformation. Considering that tumor formation is a chronic process (Grivennikov et al., 2010), a long-term model seems to be more rational for tumorigenesis investigation. As it was previously found that the promotion of cell proliferation ability is independent of intracellular location of *P. gingivalis* (Pan et al., 2014), we established an infection model.

To our knowledge, to date, only one study has established a long-term infection model which lasted for 5 weeks *in vitro*. The study found that oral cancer cells gained more aggressive capabilities and cancer stem cell properties after repeated infection by *P. gingivalis* (Ha et al., 2015). However, the direct role of *P. gingivalis* in the early stage of OSCC has seldom been focused on.

To explore the underlying effect of chronic *P. gingivalis* infection on OSCC and to identify relevant biomarkers as promising targets for therapy and prevention, we used human immortalized oral epithelial cells (HIOECs), non-tumorigenic cells which were immortalized from normal epithelial cells of oral mucosa by transfection of the HPV16 E6/E7 gene, as the cell model with no other carcinogen stimulation (Zhong et al., 2008). HIOECs were exposed to *P. gingivalis* alone at a low MOI for 5–23 weeks. During the first 15-week infection, tumorigenic properties were monitored. In order to identify gene expression and protein production changes induced by *P. gingivalis* infection, microarray and iTRAQ (isobaric tags for relative and absolute quantitation) based quantitative proteomic assays were performed using HIOECs post infection for 15 weeks (named HIOECs-Pg-15) and HIOECs alone with validation by mRNA and (or) protein levels. As infection continued, the tumorigenic properties of 23-week cells (named HIOECs-Pg-23) were also proved to be promoted. The alteration in expression of selected genes was tested as well. We found cells infected for a long time had enhanced abilities of cell proliferation, migration, and invasion. With the facilitation of bioinformatics analyses and validation, we identified some tumor-related genes, such as NNMT, FLI1, GAS6, lncRNA CCAT1, PDCD1LG2, and CD274, as the key regulators in the present tumor-like transformation model. Meanwhile, the clinical roles of some differentially expressed genes and novel proteins with potential effects on tumor-like transformation were also summarized. As a whole, we established a chronic *P. gingivalis* infection model, from which we found some candidate biomarkers that are potentially useful for monitoring in the early stage of OSCC.

## Materials and methods

### Bacteria and eukaryotic cell culture

*P. gingivalis* ATCC 33277 (American Type Culture Collection, Manassas, VA, USA) was routinely cultured (Liu et al., 2015). Briefly, *P. gingivalis* ATCC 33277 was recovered from frozen stocks on brain heart infusion (Difco Laboratories, MI, USA) agar plates supplemented with 5% defibrinated sheep's blood, 0.5% yeast, 0.1% menadione, and 1% hemin at 37°C in an anaerobic system (80% N_2_, 10% H_2_, and 10% CO_2_). Prior to use in the chronic infection model, *P. gingivalis* actively growing in broth was tested by Gram staining to ensure culture purity. The *P. gingivalis* culture was then collected by centrifugation and adjusted to an optical density of 1.0 (OD_600_) with cell culture solution without antibiotics.

HIOECs were kindly provided by Prof. Wantao Chen from Key Laboratory of Shanghai Oral Medicine, Ninth People's Hospital, Shanghai Jiao Tong University. HIOECs were cultured in Defined Keratinocyte-SFM (Gibco™, Thermo Fisher Scientific Inc., MA, USA) with growth supplement in a humidified atmosphere at 37°C (5% CO_2_) (Zhong et al., 2008).

### Long-term infection model

For long-term infection, actively growing HIOECs in 6-well plates were infected with *P. gingivalis* at an MOI of 1 for 24 h at each passage. Then infected cells were sub-cultured or fresh Defined Keratinocyte-SFM medium was replaced according to the cell growth status. Meanwhile, cell supernatants and cells lysed with sterile distilled water for 60 min from infected cells (in extra wells) were separately collected, then inoculated on agar plates with appropriate dilution and cultured under anaerobic conditions. The colonies obtained indicated that *P. gingivalis* could survive for 24 h during infection each time (data not shown). HIOECs were repeatedly infected with *P. gingivalis* for time periods of 5, 10, 15, and 23 weeks. HIOCEs without *P. gingivalis* challenge as the control were sub-cultured together with infected groups (Yu et al., 2011; Mahalingaiah et al., 2015). The method to determine the appropriate MOI for repeated infection is described in the Supplementary Material, Figure 1. Cell morphological alteration was monitored with inverted fluorescence microscopy and a transmission electron microscope.

### Cell proliferation assay by MTT

Cells (3000 cells/well in 100 μL culture medium) were seeded into 96-well plates. At each time point (24, 48, and 72 h), 3-(4,5-dimethylthiazolyl-2)-2,5-diphenyltetrazoliumbromide (MTT; Sigma-Aldrich, St. Louis, MO, USA) (5 mg/ml) was added (20 μL/well) and incubated for 4 h at 37°C. The medium was then replaced with dimethylsulfoxide (DMSO; Sigma-Aldrich, St. Louis, MO, USA) (150 μL/well), followed by vibration in darkness for 10 min until the crystal was completely dissolved (Liu et al., 2015). The optical density was detected at 490 nm with a microplate reader (Tecan, Untersbergstrasse, Austria). Defined Keratinocyte-SFM without cells was added as the blank control.

### Cell immunocytochemistry assay

Cells actively growing on sterilized glass slides were washed with PBS and fixed with 4% paraformaldehyde for 1 h at 4°C, followed by treatment with 0.1% Triton X-100 for 5 min. Antigen was retrieved with 0.1 M sodium citrate (pH 4.5) for 5 min. The monoclonal anti-Ki-67 (Zhong Shan-Golden Bridge Biological Technology Co., Beijing, China) was used as the primary antibody. Immunostaining was performed according to UltraSensitive™ S-P Immunocytochemistry and DAB substrate kit (Maixin Biotech. Co., Ltd., Fuzhou, China). A negative control without primary antibody was performed as well (data not shown).

### Colony formation assay

Well separated cells were seeded into 12-well plates (100 cells/well) and cultured for 14 days (Wu et al., 2015). The medium was replaced every 3–4 days. Cells were fixed with 4% paraformaldehyde for 1 h at 4°C, followed by staining with Giemsa working solution and photographed by stereoscopic microscope (Olympus SZX12, Japan).

### Cell cycle analysis

Cells in the exponential phase were harvested by trypsinization, fixed with 70% cold ethanol, and stored at 4°C for 16–18 h before testing. Fixed-cells were washed with ice-cold PBS, then re-suspended in propidium iodide (PI) (50 μg/mL) and RNase (100 μg/mL) mixed media for incubation at 37°C in darkness for 30 min before determination by a flow cytometer (FACS, Becton-Dickinson, Islandia, NY, USA).

### Cell migration and invasion assay

To detect the effect of long-term exposure to *P. gingivalis* on the migration and invasion abilities of HIOECs, a wound-healing assay with regular protocol (Fassi Fehri et al., 2011) and transwell system were applied, respectively. For the cell migration assay, parallel scratches were made with sterile plastic micropipette tips after cells reached 80% confluence, and photographs were taken at 0 and 24 h. Mitomycin C (4 μg/mL) was added 1 h before the assay to prevent the influence of cell proliferation. The ability of cell migration was determined by counting migrated cells at 24 h (Cai et al., 2015). For the cell invasion assay, HIOECs with and without *P. gingivalis* infection which were suspended with Defined Keratinocyte-SFM basal medium (without growth supplement) were loaded into the upper chamber of the transwell system (6.5 mm diameter, 8 μm pore size polycarbonate membrane, Corning) that was pre-coated with matrigel (BD Biosciences, CA, USA). The lower chamber was filled with Defined Keratinocyte-SFM (with growth supplement; Wang et al., 2012). After 24 h of incubation, non-invaded cells on the upper surface of the membrane were removed with a cotton swab and invaded cells on the lower surface of the membrane were fixed with methanol for 20 min, followed by staining with crystal violet (0.1%) for 20 min. The cell invasion ability was determined by counting invaded cells on the lower side.

### Gelatin zymography assay

Supernatant of HIOECs infected with *P. gingivalis* for 15 and 23 weeks as well as non-infected controls were harvested for gelatin zymography assay to detect the induction and activity of matrix metalloproteinases (MMPs). Protein concentration was adjusted with a BCA protein assay kit (Bio-Rad, Hercules, CA, USA). Supernatant samples of all groups were diluted with 2 × SDS-PAGE non-reducing buffer and separated on 8% SDS-PAGE gels containing 0.1% gelatin. The gels were incubated with 2.5% Triton X-100 for 4–6 h at room temperature, followed by another 48-h incubation with reaction buffer of the MMP Zymography Assay Kit (Applygen Technologies Inc., Beijing, China) at 37°C. The gel was stained with 5% coomassie brilliant blue R-250 and scanned (Founder Z230, Beijing, China) with clear bands against a blue background.

### Microarray and data analyses

Total RNAs of HIOECs and HIOECs-Pg-15 were extracted with an RNAiso Plus kit (TaKaRa, Dalian, China). An OE Biotech Human WT lncRNA (long non-coding RNA) Microarray (Affymetrix), which could simultaneously detect coding and non-coding genes, was conducted at the OEbiotech Corporation (Shanghai, China). Briefly, total RNA was quantified by using a NanoDrop ND-2000 (Thermo Scientific), and the RNA integrity was assessed using an Agilent Bioanalyzer 2100 (Agilent Technologies). The sample labeling, microarray hybridization, and washing were performed according to the manufacturer's standard protocols (Ambion® WT Expression Kit). The arrays were scanned by an Affymetrix Scanner 3000 (Affymetrix). Affymetrix GeneChip Command Console (version 4.0, Affymetrix) software was applied to extract raw data. Then, RMA normalization for both gene and exon level analyses was completed by Expression Console (version 1.3.1, Affymetrix) software. GeneSpring software (version 13.1; Agilent Technologies) was employed to identify aberrant gene expression analyses through fold change as well as a *P*-value calculated using Student's *t*-test. The threshold set for aberrantly regulated genes was a fold change ≥2.0 and a *P* < 0.05. Some inflammation-related and tumor-related genes were clustered with Multi Experiment Viewer (MeV, version 4.6.0). Gene ontology (GO) and Kyoto Encyclopedia of Genes and Genomes (KEGG) analysis (http://www.genome.jp/kegg/) were further applied to determine the roles mRNAs played in these GO terms or pathway enrichments. The predicted protein-protein interactions according to the tumor-related genes were analyzed with STRING10.0 (http://string-db.org/).

### iTRAQ-based quantitative proteomic assay and data analyses

HIOECs and HIOECs-Pg-15 cells were collected. Protein extraction, quality evaluation, and iTRAQ-based quantitative proteomic assay were completed at the OEbiotech Corporation (Shanghai, China). Total protein was extracted with SDT lysis buffer followed by ultrasonic treatment (100 W, 5 min) and collected by centrifugation (15,000 g, 5 min). Protein concentration was quantified with the Bradford method, and the protein quality was determined by 10% SDS-polyacrylamide gel electrophoresis. Protein (100 μg) for each sample was accurately extracted and trypsinized for 12 h at 37°C followed by vacuumization. The vacuumized peptides dissolved with TEAB (0.5 M) were labeled with a different iTRAQ reagent for 2 h at room temperature. The labeled sample was cleaned up with cation exchange chromatography using the Cation Exchange Cartridge System. Thereafter, peptides were pre-separated by Strong Cation Exchange Chromatography (SCX). Liquid chromatography-tandem mass spectrometry (LC-MS/MS) analysis was performed with Orbitrap Elite (Thermo Scientific) in Higher Energy Collisional Dissociation (HCD) mode. The raw data obtained was further analyzed with Maxquant 1.4.1.2 (Thermo Scientific), and protein false discovery rate (FDR) <0.01 was considered as the screen standard. A protein sequence database (ipi.HUMAN.v3.87.fasta) was applied for protein identification. Proteins with an expression ratio (15-week/non-infected) ≥1.5 and a *P* < 0.05 (one-tailed and equi-variance Student's *t*-test) were regarded as aberrantly regulated proteins and were clustered by R package heatmap2 software. The predicted interaction among selected proteins was analyzed with the network analysis tool Cytoscape (Cytoscape v3.3.0) (Shannon et al., 2003).

### Quantitative real-time PCR

Total RNAs of HIOECs, HIOECs-Pg-15, and HIOECs-Pg-23 were extracted and quantitative real-time PCR (q-PCR) was performed according to the manufacturer's instructions as described previously (Liu et al., 2015). The fold induction of selected genes was calculated compared with the non-infected control with the 2^−ΔΔCT^ method (Schmittgen and Livak, 2008). The nucleotide oligonucleotides sequences used are listed below.

### Western blot analysis

Whole cell protein of HIOECs, HIOECs-Pg-15, and HIOECs-Pg-23 was extracted and western blot was performed. Cells were washed with ice-cold PBS and incubated with RIPA Lysis Buffer (Beyotime Biotech. Co., Shanghai, China) containing phenylmethane-sulfonyl fluoride (1 mM) with appropriate vibration on ice for 60 min. Cells were then harvested, and the protein concentration was determined with a bicinchoninic acid protein assay kit (Beyotime Biotech. Co., Shanghai, China). An equal amount of whole cell lysate was separated with 8% SDS-polyacrylamide gel electrophoresis and transferred to a nitrocellulose filter membrane. After blocking in 5% skim milk in tris-buffered saline with 0.1% Tween (TBST) for 60 min at room temperature, the blots were probed with the following anti-rabbit primary antibodies: anti-GAS6 1:500 (Proteintech Group, Chicago, IL, USA), anti-FLI1 1:250 (Proteintech Group, Chicago, IL, USA), anti-CD274 1:500 (Abcam, Cambridge, MA, USA), anti-PDCD1LG2 1:500 (Abcam, Cambridge, MA, USA), NNMT 1:200 (Proteintech Group, Chicago, IL, USA), and GAPDH 1:1500 (Bioworld, MN, USA), at 4°C overnight. The membranes were washed with TBST three times and exposed to goat anti-rabbit IgG IRDye1 800CW secondary antibody (LI-COR, Lincoln, NE, USA) at room temperature for 1 h. The protein bands were detected with Odyssey CLX (LI-COR), and densitometric analysis was performed with Image J software (1.42q).

**Table:** 

Gene	Forward primer	Reverse primer
*CXCL10*	TTGAAATTATTCCTGCAAGCC	TCAGACATCTCTTCTCACCCTTC
*CSF1*	TGCAGGAACTCTCTTTGAGGCTGA	TCCAGCAACTGGAGAGGTGTCTCATA
*IL6*	ACTCACCTCTTCAGAACGAATTG	GCCATCTTTGGAAGGTTCAGGTTG
*NNMT*	TCCTTTAAGGAGATCGTCGTCAC	GCTTGACCGCCTGTCTCAAC
*CYGB*	CTTCAGCCAGTTCAAGCACA	AAGTACACCGGTTCCACCTTG
*CXCL11*	GACGCTGTCTTTGCATAGGC	GGATTTAGGCATCGTTGTCCTTT
*FLI1*	CAGTCGCCTAGCCAACCCTGG	GGGCAATGCCGTGGAAGTCAAAT
*WFDC2*	TGCCCCCAGGTGAACATTAAC	CCATTGCGGCAGCATTTCAT
*GAS6*	TGCTGTCATGAAAATCGCGG	GCATGTAGTCCAGGCTGTAGA
*TGM1*	GATCGCATCACCCTTGAGTTAC	CCGCAGGTTCAGATTCTGCCC
*TRIM29*	GCACCGGACACCATGAAGAGA	AGGAGACGAGGGCTGGTATGA
*GRHL3*	GCAAGCGAGGAATCTTAGTCAA	ACGTGGTTGCTGTAATGCTGA
*CCAT1*	TCACTGACAACATCGACTTTGAAG	GGAGAAAACGCTTAGCCATACAG
*PKP1*	GACCAGGACAACTCCACGTT	CTGCTGGTGGTCCCATAGTT
*CD274*	CATCTTATTATGCCTTGGTGTAGCA	GGATTACGTCTCCTCCAAATGTG
*PDCD1LG2*	CAACTTGGCTGCTTCACATTTT	TGTGGTGACAGGTCTTTTTGTTGT
*GAPDH*	GAAGGTGAAGGTCGGAGTC	GAAGATGGTGATGGGATTTC

### Statistical analysis

Non-parametric tests were applied for the statistical analysis. Comparisons within different groups in general were analyzed with the Kruskal-Wallis H test. *P* < 0.05 was considered statistically significant. Differences between each of two groups were analyzed with the Mann-Whitney U-test. *P* < α′ was considered statistically significant; α′ was calculated for multiple testing correction. α′ = α/0.5*p* (*p* – 1) (α = 0.05, *p* represents the number of groups). The statistical analysis for microarray and proteomic assays was as described above. SPSS 17.0 software package (IBM, Armonk, NY, USA) was used.

## Results

### Cell model establishment and morphological alteration

**Figure 1:**
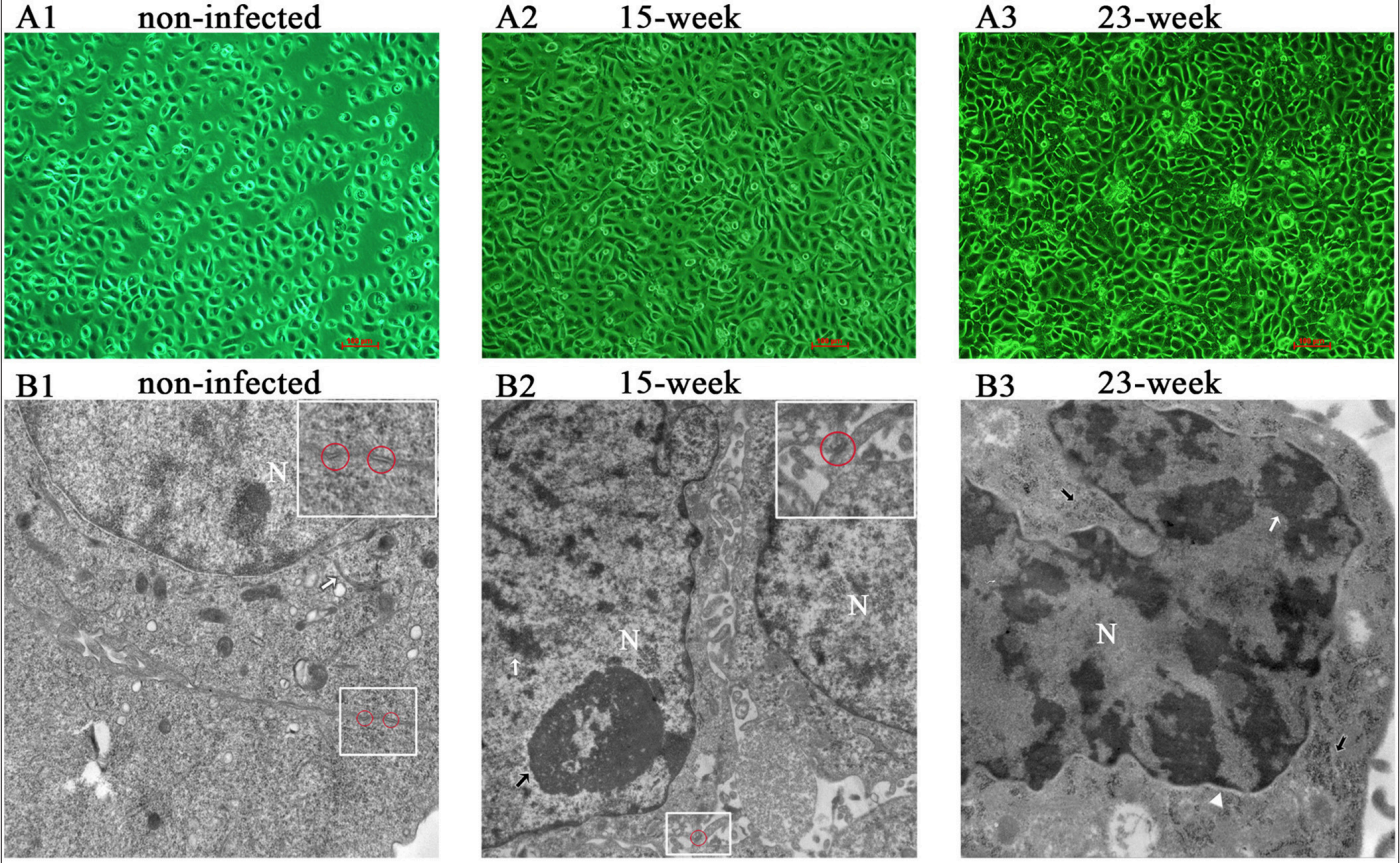
**Cell morphological change after long-term exposure to ***P. gingivalis***. (A)**. Morphological differences among HIOECs (non-infected), HIOECs-Pg-15 (15-week), and HIOECs-Pg-23 (23-week) were shown with inverted microscopy. HIOECs presented regular cobble-stone-like shape with obvious contact inhibition (**A1** × 100) while infected cells were overlapped and showed irregular shape with more stretched cells (**A2, A3** × 100). **(B)**. Transmission electron microscopy showed the ultrastructure alteration. HIOECs showed regular nuclear/cytoplasmic ratio with rich and thick tonofilaments (white arrow) and multiple organelles (B1 × 20,000). The cellular junction between HIOECs was strong and composed of multi-desmosomes (red circle, partially magnified details at the top right corner, **B1**). HIOECs-Pg-15 and HIOECs-Pg-23 showed increased nuclear/cytoplasmic ratio, clearly abundant euchromatin and relatively simple organelles **(B2,B3)**. The cellular junction of HIOECs-Pg-15 was weakened while few desmosomes were occasionally seen (red circle, partially magnified details at the top right corner, B2 × 10,000). The nucleoli and heterochromatin are respectively marked with black and white arrows **(B2)**. The nucleus of HIOECs-Pg-23 became more deformed while obvious heterochromatin margination (indicated with a white arrow) was seen (B3 × 20,000). As shown with a white triangle, the perinuclear space was expanded **(B3)**. Abundant ribosomes are shown with black arrows **(B3)**.

According to the result of preliminary experiments (Supplementary Material, Figure 1), a long-term infection model was successfully established by insulting HIOECs with *P. gingivalis* at the MOI of 1 for the first 5–15 weeks and then another 8 weeks (23 weeks in total). HIOECs grew regularly with normal contact inhibition (Figure 1A1). On the contrary, HIOECs-Pg-15 and HIOECs-Pg-23 presented in slender or other anomalous shapes with a trend toward absent contact inhibition (Figures 1A2,A3). Interestingly, the irregular cell shape was more obvious with infection over time. We further observed the alteration of cellular ultrastructure under a transmission electron microscope (Figures 1B1–B3). The rich and thick tonofilaments, which are typical in well-differentiated HIOECs (Figure 1B1), were hardly found in infected cells (Figures 1B2,B3). The cell junction in infected cells became weakened, and the desmosome could only be occasionally detected (Figure 1B2). Furthermore, the nuclear cytoplasmic ratio was increased with obvious heterochromatin margination (Figure 1B3).

### Long-term exposure to *P. gingivalis* promoted cell proliferation

**Figure 2:**
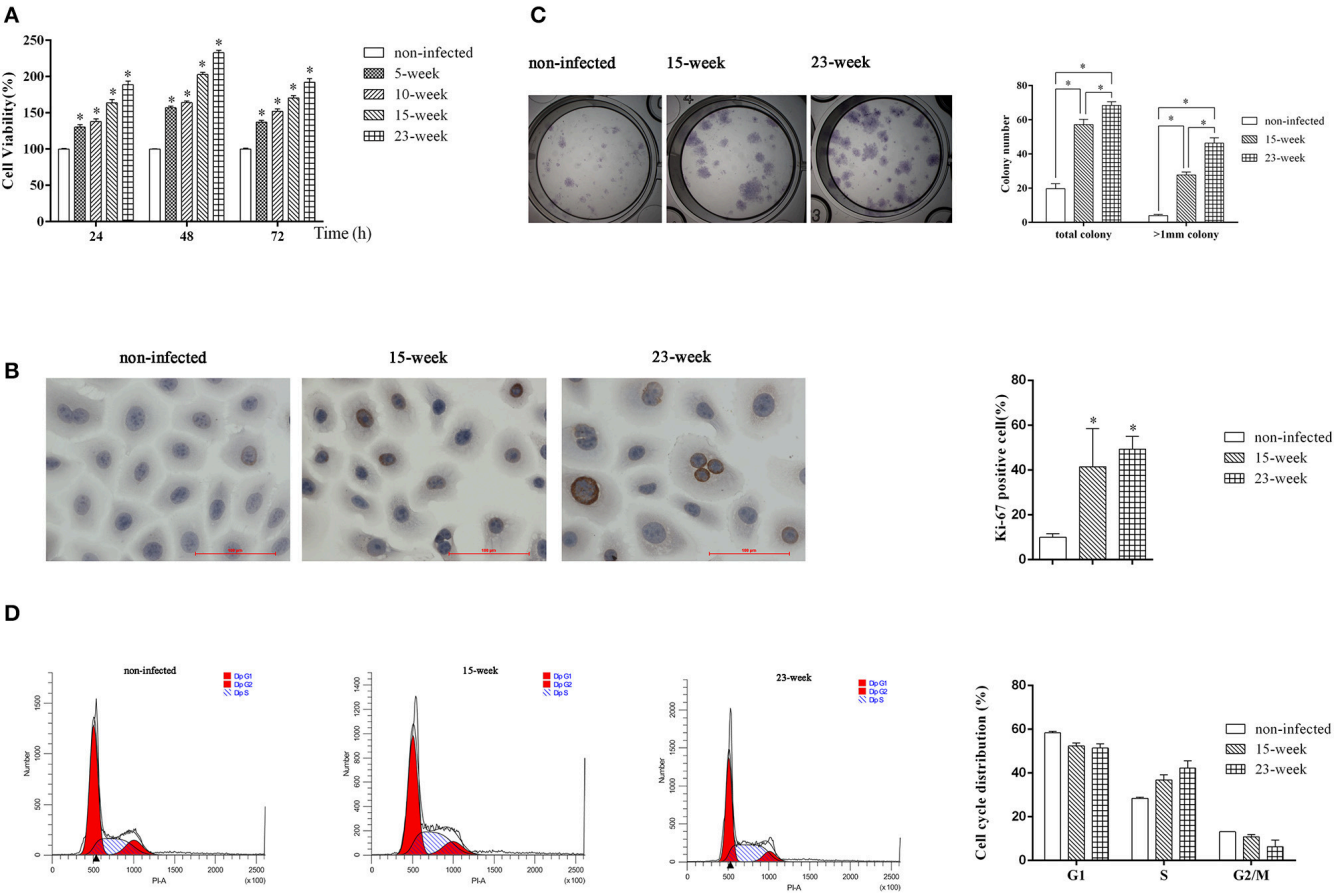
**Effect of long-term exposure to ***P. gingivalis*** on cell proliferation and cell cycle**. In the long-term infection for up to 23 weeks' duration, cells were infected with *P. gingivalis* at an MOI of 1 for 24 h every 3 or 4 days. **(A)**. The non-infected control and cells consistently exposed to *P. gingivalis* for 5, 10, 15, and 23 weeks were collected and the proliferation ability was determined. The data are presented as the mean ± standard deviation of three independent assays and analyzed with Kruskal-Wallis H test post Mann-Whitney U test. ^*^Significant difference (*P* < 0.005) compared with cells of non-infected control **(B)**. To determine the expression or Ki-67 after long-term infection, HIOECs (non-infected), HIOECs-Pg-15 (15-week), and HIOECs-Pg-23 (23-week) were used for immunohistochemistry assay (×400). The percentage of Ki-67 positive staining cells was calculated in ≥5 microscopic fields per slide from three independent experiments. Data are shown as the mean ± standard deviation and analyzed with Kruskal-Wallis H test post Mann-Whitney U test. ^*^Significant difference (*P* < 0.0167) compared with cells of the non-infected control. **(C)**. For colony formation assay, cells were cultured for 2 weeks and stained with Giemsa working solution. Total colonies and colonies with diameter >1 mm were separately calculated from three independent experiments. The data are presented as the mean ± standard deviation and analyzed with Kruskal-Wallis H test post Mann-Whitney U test. ^*^Significant difference (*P* < 0.0167) compared between different groups. **(D)**. The effect of long-term exposure to *P. gingivalis* on cell cycle distribution was determined by flow cytometry and analyzed with ModFit software. Cells were collected and measured by flow cytometry. Cells with DNA content of 2N, 2N to 4N, and 4N represent the G1, S, and G2/M phase populations, respectively.

We first performed an MTT assay and found that the proliferation ability of infected HIOECs (5–15 weeks) was significantly increased (*P* < 0.005) compared with the non-infected control (Figure 2A). When the infection model was continued for another 8 weeks, HIOECs-Pg-23 also showed greater proliferation ability compared with the non-infected control (*P* < 0.005; Figure 2A).

We further performed an immunocytochemistry assay to compare expression of Ki-67, a cellular marker of malignancy, among HIOECs, HIOECs-Pg-15, and HIOECs-Pg-23. The positive staining was clearly located in the nuclei of infected cells. The percentages of Ki-67 positive staining in HIOECs-Pg-15 and HIOECs-Pg-23 were significantly higher than that of the non-infected control (*P* < 0.0167; Figure 2B). Remarkably, some of the cells infected with *P. gingivalis* showed more obvious mitoses, which is an important feature commonly identified in malignant neoplasms.

To investigate the proliferative ability of the single cell, we performed a colony formation assay. HIOECs-Pg-15 and HIOECs-Pg-23 formed many more total colonies, including colonies larger than 1 mm in diameter, with a significant difference (*P* < 0.0167) compared with HIOECs. The same trend was also found between the treatment groups (*P* < 0.0167; Figure 2C).

### Long-term exposure to *P. gingivalis* accelerated cell cycle

S phase fraction represented by the cell percentage in the S phase is known as an important measure of tumor proliferative activity. Obviously, the number of S phase cells is higher in HIOECs-Pg-15 and HIOECs-Pg-23 than that in HIOECs (28.40 ± 0.56%). The S phase percent of HIOECs-Pg-23 (42.25 ± 3.41%) was higher than that of HIOECs-Pg-15 (36.85 ± 2.30%) (Figure 2D). Thus, the S phase fraction of HIOECs-Pg-15 and HIOECs-Pg-23 was higher than that of HIOECs.

### Long-term exposure to *P. gingivalis* promoted cell migration and invasion abilities

**Figure 3:**
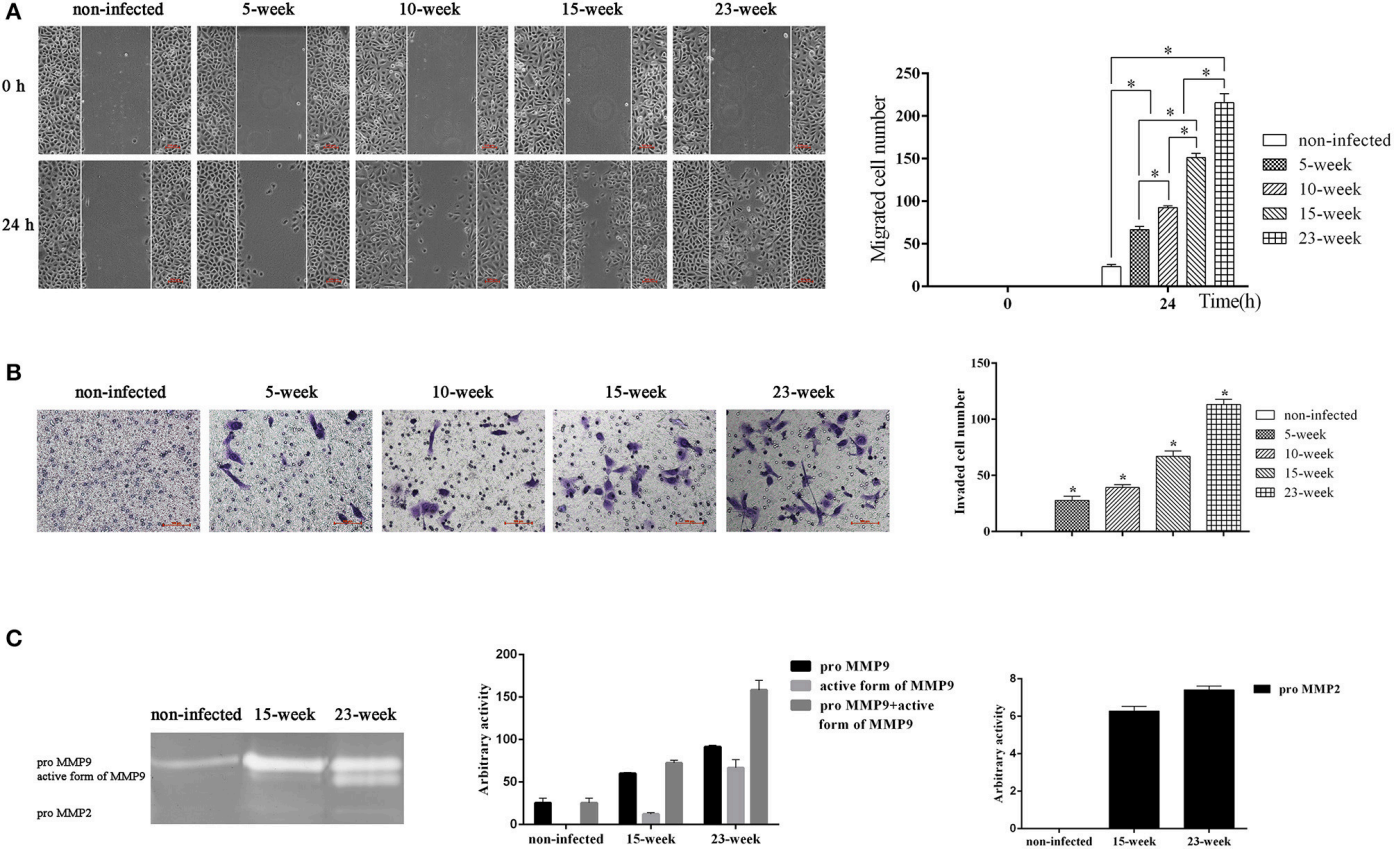
**Effect of long-term exposure to ***P. gingivalis*** on cell migration and invasion. (A)**. Wound-healing assay was applied to determine the ability of cell migration in different groups. Parallel scratches were made at 0 h when cells reached 80% confluence. Cells in different groups were cultured for 24 h and photographed at 0 h and 24 h (×100). Cells that migrated were counted from three independent assays. Data are shown as the mean ± standard deviation and analyzed with Kruskal-Wallis H test post Mann-Whitney U test. ^*^Significant difference (*P* < 0.005) compared between different groups. **(B)**. Cells from different infected groups together with the non-infected control were collected, and a transwell system was used for cell invasion assay. To compare the invasion ability of each group, invaded cells were counted in five microscopic fields per well from three independent assays. Data are shown as the mean ± standard deviation and analyzed with Kruskal-Wallis H test post Mann-Whitney U test. ^*^Significant difference (*P* < 0.005) compared with cells of the non-infected control **(C)**. After cells were consistently infected with *P. gingivalis* for 15 and 23 weeks, cell supernatant was collected together with that of the non-infected control to determine the activity of MMP9 and MMP2 by gelatin zymograph. Enzyme activities are expressed from densitometric analysis with arbitrary units by applying Image J software (1.42q). Data are shown as the mean ± standard deviation from three independent assays.

The ability of cells to migrate and invade could eventually induce metastatic proliferation in distant organs. With the application of a wound-healing assay, we found that the number of migrated cells was significantly increased in groups infected with *P. gingivalis* compared with the non-infected group (*P* < 0.005). Within the treatment groups, the migration ability of four groups was gradually increased (*P* < 0.005; Figure 3A).

To evaluate the invasion ability of HIOECs with and without *P. gingivalis* infection, a transwell system with pre-coated matrigel was used. After incubation for 24 h, non-infected cells showed no invasion while cells infected with *P. gingivalis* showed great ability to invade through the matrigel and they were detected on the down-side of the membrane (*P* < 0.005; Figure 3B).

We further investigated the activity of MMP2 and MMP9 in culture supernatant (Inaba et al., 2014). As shown, the amount of proMMP9 secreted by HIOECs-Pg-15 was significantly increased compared with the non-infected control. After infection for another 8 weeks, the amount of the active form of MMP9 secreted by HIOECs-Pg-23 was much higher than that secreted by HIOECs-Pg-15. Additionally, the secretion of proMMP2 could only be detected with *P. gingivalis* infection (Figure 3C).

### Differential bioinformatics analyses between HIOECs and HIOECs-Pg-15

After the first 15-week infection when HIOECs had already performed relatively obvious tumor biological alteration, microarray- and iTRAQ-based quantitative proteomic assay were applied to explore the possible regulators involved in the present tumor-like transformation model. Chronic infection by *P. gingivalis* was continued for another 8 weeks as we attempted to investigate further tumorigenic change.

**Figure 4:**
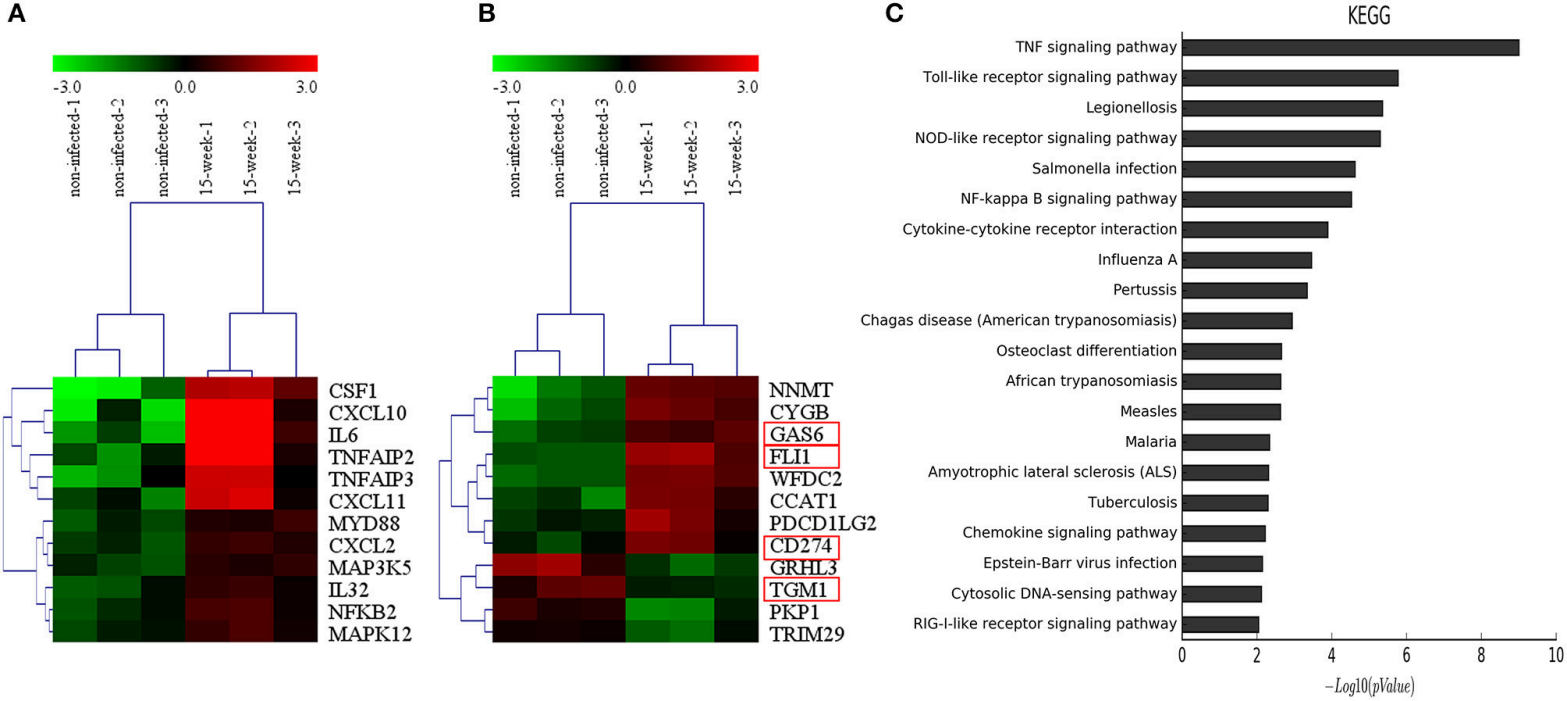
**Microarray analysis. (A,B)**. Based on microarray data, inflammation-associated and tumor-associated genes were clustered and shown in tree view with mRNA expression from low (green) to high (red). **(C)**. KEGG pathways analysis revealed that genes selected were involved in several key pathways such as TNF signaling, Toll-like receptor signaling and NF-kappa B signaling, which were closely associated with regulation of tumor development under an inflammatory microenvironment.

According to the microarray data, 471 genes were up-regulated while 191 genes were down-regulated. Genes associated with inflammation (Figure 4A) and tumor (Figure 4B) were clustered, respectively. KEGG pathways analysis (Figure 4C) and GO analysis (Supplementary Material, Figure 2) were performed on the selected genes. KEGG pathways analysis revealed that *P. gingivalis* may activate some classic pathways involved in inflammation-associated tumorigenesis, such as TNF, Toll-like receptor, and NF-kappa B signaling pathways. Some differentially expressed genes were summarized based on their clinical functions as biomarkers. As shown, NNMT, FLI1, SLPI, LAMP3, SERPINA1, GAS6, and CD274 have been often used as biomarkers for OSCC or head and neck cancer, while other genes listed have seldom been investigated in oral cancer and may be considered as novel biomarkers for OSCC prevalence and treatment (Supplementary Material, Table 1).

**Figure 5:**
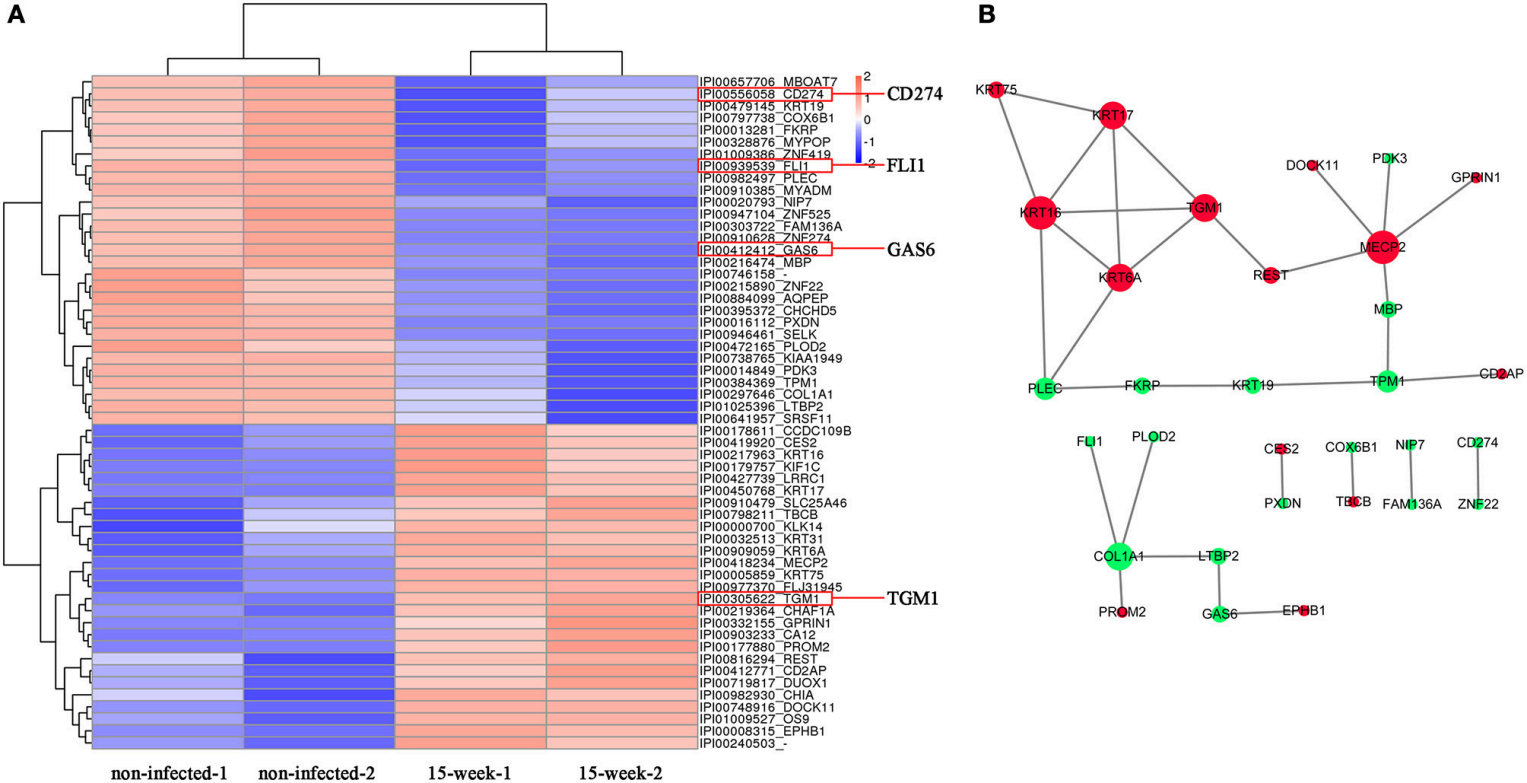
**Proteomic analysis. (A)**. Based on proteomic analysis, 56 proteins identified with aberrant expression were clustered and shown in the heatmap from high (orange) to low (blue) expression **(B)**. The predicted protein-protein interactions are listed while up-regulation is shown in red and down-regulation shown in green.

Based on the 5,173 proteins detected from proteomic assay, 56 proteins were differentially expressed (Figure 5A) and the potential interaction among these proteins is shown (Figure 5B). Comparing with the results of the microarray assay, notably, FLI1, GAS6, CD274, and TGM1 were also aberrantly expressed at the protein level. iTRAQ is believed to be a high-throughput method, which significantly enhances the reliability and degree of coverage of protein identification as multi-peptides are quantified (Ross et al., 2004). Using iTRAQ-based proteomic assay, we found 10 novel proteins that may be potentially associated with tumorigenesis (Supplementary Material, Table 2).

### Validation of microarray and proteomic assay

**Figure 6:**
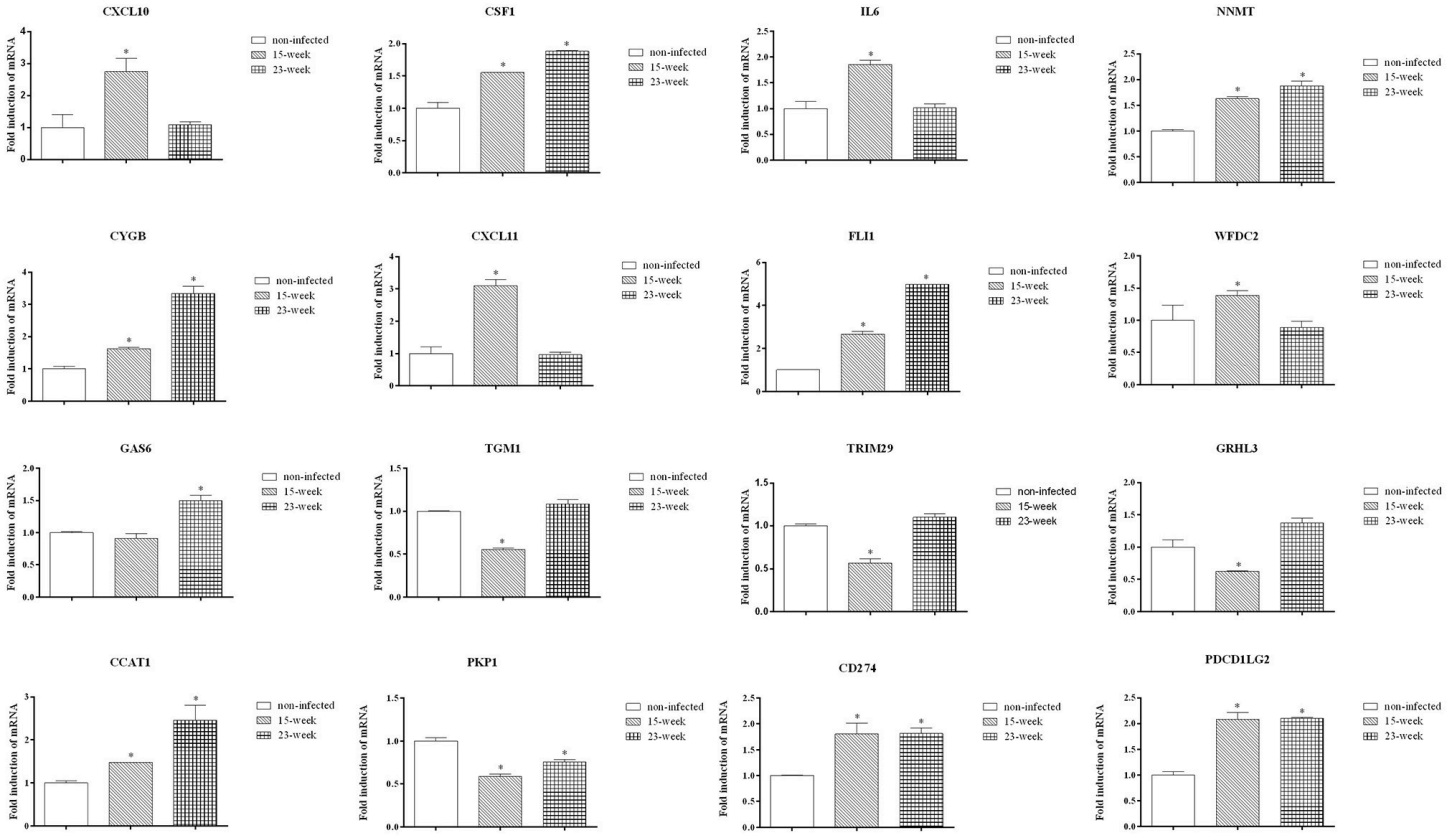
**Validation by quantitative real-time PCR**. To validate the mRNA expression of selected genes, quantitative real time PCR was applied. Data were normalized to GAPDH mRNA, and the fold inductions of selected genes were calculated relative to cells without infection. Data are shown as mean ± standard deviation and were analyzed with Kruskal-Wallis H test post Mann-Whitney U test. ^*^Significant difference (*P* < 0.0167) compared with cells of the non-infected control.

Data validation of the microarray assay was first performed using q-PCR. Some genes were selected from Figures 4A,B. As shown, CXCL10, CSF1, IL6, NNMT, CYGB, CXCL11, FLI1, WFDC2, CCAT1, CD274, and PDCD1LG2 were up-regulated while TGM1, TRIM29, GRHL3, and PKP1 were down-regulated on HIOECs-Pg-15 (*P* < 0.0167; Figure 6), which is in correspondence with the microarray data (Supplementary Material, Table 1). Interestingly, comparing with non-infected cells, the mRNA expression of CSF1, NNMT, CYGB, FLI1, GAS6, CCAT1, CD274, and PDCD1LG2 was increased, while that of PKP1 was decreased in HIOECs-Pg-23 (*P* < 0.0167; Figure 6).

**Figure 7:**
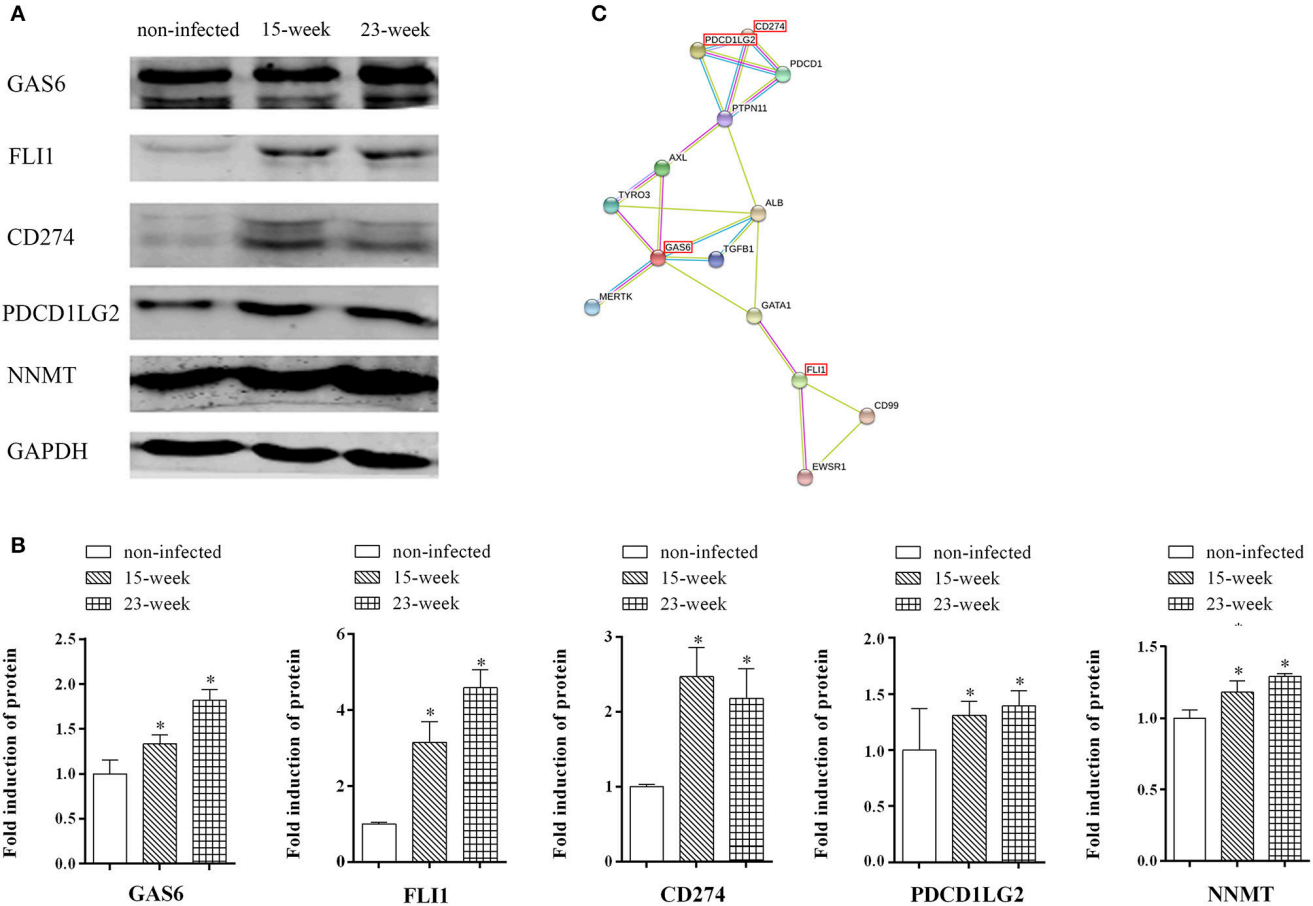
**Key factors involved in the present infection model. (A,B)**. Whole cell lysates of HIOECs, HIOECs-Pg-15, and HIOECs-Pg-23 were harvested for western blot assay. The expression of tested proteins was normalized to GAPDH expression with densitometric analysis. Data are shown as mean ± standard deviation and analyzed with Kruskal-Wallis H test post Mann-Whitney U test. ^*^Significant difference (*P* < 0.0167) compared with cells of the non-infected control. **(C)**. The protein-protein interaction among key regulators was predicted.

Western blot assay was done to further confirm the findings of both the microarray and quantitative proteomic assay. The increased protein production of FLI1, GAS6, and CD274, which were up-regulated in both microarray and proteomic data, can be seen in the western blot assay (Figures 7A,B) indicating that the results of the western blot were consistent with those from microarray and proteomic analyses. Also, the protein level of PDCD1LG2, an important paralog of CD274, was validated by western blot. Confirmed by western blot again, NNMT was up-regulated in *P. gingivalis* infected cells compared with the normal control (Figures 7A,B). The predicted protein interactions among NNMT, FLI1, GAS6, PDCD1LG2, and CD274 were specifically analyzed (Figure 7C).

**Figure 8:**
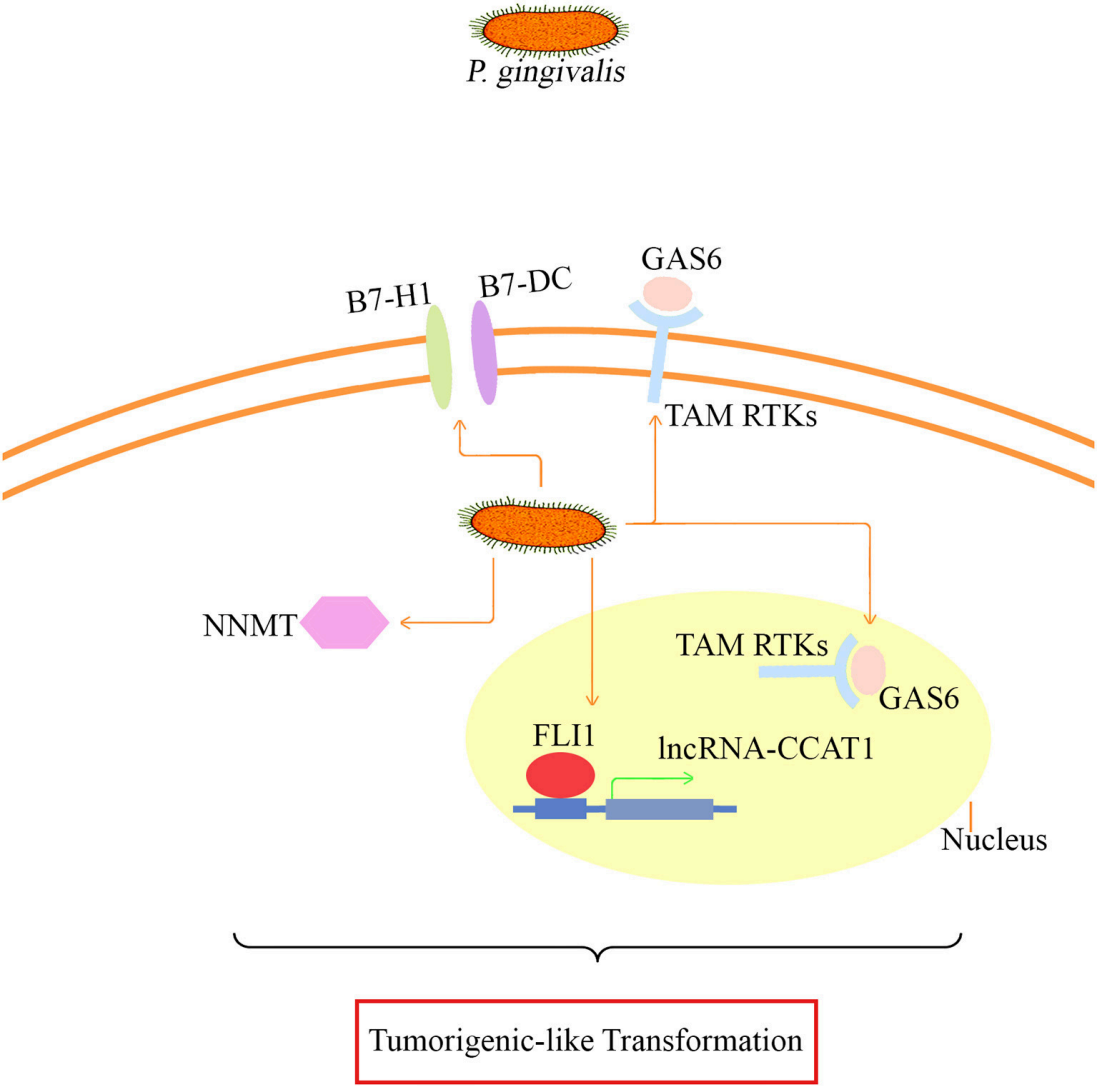
**The proposed mode pattern of tumorigenic-like transformation mechanism based on the present model**. Under long-term infection, *P. gingivalis* may activate key factors such as B7-H1/B7-DC (also known as CD274/PDCD1LG2 based on our microarray and proteomic assay data), GAS6 that binds to TAM RTKs, CCAT1/FLI1, and NNMT for the eventual tumorigenesis transformation. The potential interaction of the presented key factors and the specific regulation pathways needs further investigation.

Taken together, NNMT, FLI1, GAS6, lncRNA CCAT1, PDCD1LG2, and CD274, whose differential expression was confirmed, may be involved in the major mechanism of tumor-like transformation induced by chronic *P. gingivalis* infection. The proposed mode pattern is shown (Figure 8). To date, we can only suppose that chronic *P. gingivalis* infection might regulate cell tumor biological alteration by activation of the above genes. However, the potential interaction of these key factors and the regulation of pathways specific to each factor needs further investigation.

## Discussion

*P. gingivalis*, with up to 85% detection rate in periodontal disease sites (Yang et al., 2004), can invade into and exit from epithelial cells with proper control of its population for persistent colonization and chronic infection (Sakanaka et al., 2016). By establishing the present infection model where HIOECs were chronically challenged with *P. gingivalis* for up to 23 weeks, we found that *P. gingivalis* significantly increased the tumorigenic properties of HIOECs by promoting proliferation, migration, and invasion capabilities with cell morphological alteration. Further, some genes involved in inflammation and tumor development were found to be regulated. These findings were consistent with our hypothesis.

To our knowledge, this infection model lasted for the longest time ever reported for the tumor-promoting effect of *P. gingivalis* on non-tumorigenic cells *in vitro*. The proliferation ability of HIOECs was found to be promoted as we expected, similar to other findings (Mao et al., 2007; Kuboniwa et al., 2008; Binder Gallimidi et al., 2015). With the facilitation of a transmission electron microscope, infected cells showed similar ultrastructure to the characteristics of typical cancer cells. Few desmosomes of infected cells could be found and the cell junction became weakened (Figure 1B2), which might signify a precondition for the migration and invasion abilities of infected-HIOECs. Plakophilin 1 (PKP1) is a key molecule for desmosome development and stability, which plays an important role in cell-cell contact. Of note, a recent global proteomic analysis revealed that the reduction of PKP1 was found to be associated with poorer disease-specific survival and significantly shorter time to onset of distant metastasis in OSCC patients (Harris et al., 2015). Thus, we suppose that down-regulation of *PKP1* induced by *P. gingivalis* may enhance cell metastasis during tumor development. Other molecules which were involved in cell migration and invasion as well as metastasis are MMP2 and MMP9. It was reported that *P. gingivalis* promoted invasive and metastasis abilities of OSCC cells. *P. gingivalis* was found to induce proMMP9 production at an MOI of 1 for up to 24 h and to activate the proenzyme of MMP9 by activating p38/HSP27, ERK1/2-Ets1, and proteinase-activated receptor 2 (PAR2)/NFκB pathways (Inaba et al., 2014). In the current study, chronic infection with *P. gingivalis* increased the proMMP9 amount and its activation, which is consistent with the findings of others. However, the inhibiting effect of *P. gingivalis* in cell migration has also been reported (Laheij et al., 2013, 2015), which is in contrast to our findings. Rather than a long-term infection model, it was found that *P. gingivalis* infection for 17 h at an MOI of 10, 100, and 1,000 induced delayed wound healing of immortalized oral epithelial cells, which originated from squamous cell carcinoma tissues and demonstrated great migratory ability after 17 h. The effect of *P. gingivalis* on cell migration might vary from different cell models with different infection degrees and cell origins. Moreover, differences in infection duration might lead to diverse results. Based on the present model, we focused on the chronic infection role of *P. gingivalis* and demonstrated that *P. gingivalis* could effectively promote cell migration.

With regard to the basis of tumor biological transformation, the eventual malignant transformation could not be concluded as cells infected for up to 23 weeks still failed to form colonies in soft agar (data not shown). Further, transformation may be explored by establishing an infection model for a longer duration. Besides, smoking and alcohol also contribute to OSCC development (Kudo et al., 2016). As the inter-communications among oral pathogens are rather complicated (Atanasova and Yilmaz, 2015), other pathogens such as *F. nucleatum*, whose regulating role in inflammation and cancer was found in the lower gastrointestinal tract, may be introduced to investigate multispecies influence on cell malignant transformation (Bashir et al., 2016).

The immortalized epithelial cells generated by overcoming senescence and acquiring immortality have been widely used as an important tool for tumorigenesis investigation (Moffatt-Jauregui et al., 2013). As reported, *H. pylori* could induce malignant transformation of immortalized human gastric epithelial cells with infection for 45 days (Yu et al., 2011). Similarly, chronic exposure to H_2_O_2_ in low concentrations for 6 months could lead to malignant transformation of immortalized human kidney epithelial cells (Mahalingaiah et al., 2015). In comparison with primary cultured epithelial cells with a finite lifespan lasting for around nine passages, the immortalized cell model in which cells have gained the ability of immortalization provides a precondition for the successful establishment of long-term infection. However, at the same time, the already acquired immortalized ability, which is identified as the first step of cell malignant transformation, may partly weaken the effect of *P. gingivalis* on tumorigenic properties in the present model. As study continues, primary epithelial cells should also be applied in establishment of the malignant transformation model. Specifically, to maximally weaken the effect of donor-to-donor variability from the primary cells, cells should be collected from various donors if possible (Moffatt-Jauregui et al., 2013).

An inflammatory microenvironment is an indispensable component of all types of tumors (Grivennikov et al., 2010). In our study, some inflammatory factors were up-regulated in both microarray and q-PCR data (Figures 4A, 6). For example, colony stimulating factor 1 (CSF1), which can stimulate cytokine and protease secretion, is involved in TNF signaling, a typical tumor promoting signaling pathway (Figure 4C; Grivennikov et al., 2010). The overexpression of CSF1 has also been found in various cancers in paracrine or autocrine ways (Azzam et al., 2013; Richardsen et al., 2015; Dang et al., 2016). Within the inflammatory microenvironment, ROS is another key factor closely related to tumor development (Grivennikov et al., 2010). In the present study, the mRNA of cytoglobin (CYGB) was found to be increasingly elevated during persistent infection. CYGB is a member of the globin family which functions as an important mediator under oxidative stress and is well known to scavenge ROS in order to protect host cells from injury triggered by oxidative stress. The increased expression of CYGB detected in our study showed the dynamic dialogue that is occurring between the chronic *P. gingivalis* infection and host cells in the context of host oxidative stress regulation which in turn can negatively impact the normal physiology and (or) ATP metabolism of cells. The cytoprotective effect of CYGB may cause carcinogenesis or aggressiveness as an oncogene in certain types of tumor including head and neck cancer (Shaw et al., 2009), which is consistent with our findings. The ability of *P. gingivalis* to modulate ROS as demonstrated before (Choi et al., 2013; Hung et al., 2013) during the infection could also contribute the observed cell pro-survival phenotype in the present study and the disturbance in the cell homeostasis.

With the application of microarray, we summarized the clinical role of genes with aberrant expression which may be applied as useful biomarkers for OSCC (Supplementary Material, Table 1). Some novel proteins that may be involved in tumorigenesis were also detected with proteomic assay (Supplementary Material, Table 2). For instance, PDK3 and CCDC109B, which are both located in mitochondria and are closely related to energy metabolism, are respectively involved in ROS generation and cell death pathway activation. ZNF419 and MYPOP are nucleus-located proteins that related to transcription. SRSF11 and NIP7, another two proteins located in nucleus, are related to pre-mRNA and pre-rRNA processing, respectively. The tumorigenic roles of these proteins remain unknown and needs to be investigated in the future.

Interestingly, we identified the up-regulation of CCAT1 (Colon cancer associated transcript 1) in microarray data validated by q-PCR, and this is the first time that CCAT1 is reported to be related to oral epithelial cells. CCAT1 is a newly recognized lncRNA that is up-regulated in various cancers (He et al., 2014; Ma et al., 2015; Zhang et al., 2015; Zhao et al., 2015; Zhu et al., 2015; Li et al., 2016) and is associated with tumor cell migration and proliferation. It has been reported that CCAT1 was activated through binding to its promoter region by c-Myc, the transcriptional factor, in order to promote cell proliferation and invasion to the surrounding tissues (He et al., 2014). Whether *CCAT1* is up-regulated in oral cancer and its possible role in cancer development requires further investigation. Friend leukemia virus integration 1 (FLI1) is another important transcriptional factor which was co-expressed with CCAT1 in our study. To analyze if FLI1 could also regulate the expression of CCAT1, we found the possible binding site of FLI1 in the promoter region of CCAT1 (http://www.ensembl.org/, http://jaspar.genereg.net/). In future studies, we will be focusing on whether FLI1 indeed regulates the transcription of CCAT1 and how FL11-regulated CCAT1 functions on tumor-like transformation of HIOECs induced by *P. gingivalis*.

Another up-regulated gene of interest which was confirmed at mRNA and protein levels is Nicotinamide N-methyltransferase (NNMT). NNMT is an enzyme closely related to biotransformation and is highly expressed in various malignances (Sartini et al., 2012). The up-regulation of NNMT was also detected in OSCC patients, which may be applied as a potential biomarker for early diagnosis (Sartini et al., 2012). Remarkably, in a very recent report, the overexpression of *NNMT* was found in cancer stem cells (CSCs)-enriched subpopulation in head and neck cancer (Pozzi et al., 2015). Chronic *P. gingivalis* infection on oral cancer cells has been reported to induce more aggressive transformation by acquisition of CSCs properties (Ha et al., 2015). Thus, it is possible that the increased expression of NNMT may be also associated with OSCC development.

Combined techniques of microarray and proteomic data analyses lead us to discover the genes (such as FLI1, GAS6, and CD274) that may be involved in the tumorigenesis of HIOECs after chronic *P. gingivalis* infection. CD274 (also known as B7-H1) and its paralog, PDCD1LG2 (programmed cell death 1 ligand 2, also known as B7-DC), are types of transmembrane protein normally expressed on immunocytes, which function in preventing autoimmunity under infected conditions. Within the inflammation-tumor microenvironment, CD274 and PDCD1LG2 play an important role in facilitating immune escape for tumor cells. Also, according to recent studies, they were overexpressed in various types of solid tumors including OSCC and were highly associated with distant metastasis and poor prognosis (Konishi et al., 2004; Ohigashi et al., 2005; Tsushima et al., 2006; Thompson et al., 2007). Similarly with our findings, the overexpression of CD274 and PDCD1LG2 in oral cancer cells can be induced by *P. gingivalis* after 24 h of infection at an MOI of 100 (Groeger et al., 2011). In particular, the regulation of CD274 and PDCD1LG2 in oral cancer cells induced by *P. gingivalis* was recently reviewed as a part of important mechanisms involved in the association between oral bacteria and cancer, which was consistent with our findings (Hajishengallis, 2015). Growth arrest specific 6 (GAS6) is one of the important bridging ligands of TAM (TYRO3, AXL, and MERTK) RTKs (receptor tyrosine kinases), participating in activation of TAM RTKs and enhancement of cell transformation (Graham et al., 2014). GAS6 is regarded as a crucial factor in regulating cell proliferation. The endogenous expression of *GAS6* contributes to maintenance of cancer stem cells (Jung et al., 2016). Overexpression of *GAS6* can promote migration and invasion of oral cancer cells (Jiang et al., 2015). Of note, within the inflammation microenvironment, the expression level of GAS6 is significantly elevated in response to M-CSF (also known as CSF1; Loges et al., 2010). FLI1 was identified as a hallmark of Ewing sarcoma by a mechanism of EWS-FLI1 fusion oncoprotein (Song et al., 2015) and an oncogenic regulator in malignant phenotype promotion (Zhang et al., 2011). In Ewing tumorigenesis, EWS-FLI1 can induce G1/S regulatory genes such as cyclin D and cyclin E and inhibit tumor suppressor genes such as p21 and p57^kip^, these data are similar to what we have found in our previous study (Dauphinot et al., 2001; Matsumoto et al., 2001; Liu et al., 2015). In addition, up-regulation of FLI1 could activate the Rho GTPase pathway associated with breast cancer metastasis (Song et al., 2015). As OSCC is a common type of tumor with high risk and often evades detection until metastasis to cervical lymph nodes with a poor prognosis (Severino et al., 2015), thus, prompt detection is of importance. Based on our study, we suppose that chronic *P. gingivalis* infection may regulate cell migration and invasion through FLI1 activation. The role of FLI1 as a potential prognostic marker in OSCC requires investigation in a large scale epidemiological study. Except for the key regulators mentioned above, other aberrantly expressed genes from the microarray were not identified by proteomic assay. Three reasons might contribute to this variance. Firstly, some genes were secretory, such as some inflammatory factors, while the protein sample used for the proteomic assay was extracted from cells rather than the culture supernatant. Secondly, *P. gingivalis* can produce proteolytic enzymes such as collagenases and gingipains, which may hydrolyze proteins. Thirdly, *P. gingivalis* could modulate protein expression by post-translation modification such as lysine acetylation (Butler et al., 2015).

Overall, we confirmed the active role of *P. gingivalis* in tumor-like transformation with long-term infection. The bioinformatics analyses provided us a novel insight into some candidate biomarkers with application potential in the early stage of OSCC. Based on the present model, *P. gingivalis* performed as a motivator in tumor-like transformation under a certain inflammatory microenvironment. For further study, animal models should be applied to explore the role of chronic *P. gingivalis* infection in oral tumor while the possible molecular regulators should be further validated. Since *P. gingivalis* can not only be detected in tissues of periodontitis but also present in other parts of the human body, such as the respiratory (Tan et al., 2014) and vascular (Yamaguchi et al., 2015) and esophagus (Gao et al., 2016) tissues, we would pay more attention to the possible effect of *P. gingivalis* as the biomarker for various types of tumors.

## Author contributions

YP, JL, HW, and FG designed the study. FG performed the experiments with the help from YP, YG, JL, and FG wrote the final manuscript. YP, HW, CL, YG, and HZ revised the manuscript.

## Funding

This study was supported by National Natural Science Foundation of China (81470745).

### Conflict of interest statement

The authors declare that the research was conducted in the absence of any commercial or financial relationships that could be construed as a potential conflict of interest.
