# Supplementary material for: Persistent Exposure to Porphyromonas gingivalis Promotes Proliferative and Invasion Capabilities, and Tumorigenic Properties of Human Immortalized Oral Epithelial Cells
Source: Front Cell Infect Microbiol. 2017 Feb 24;7:57. doi: 10.3389/fcimb.2017.00057 (PMC5323389; doi:10.3389/fcimb.2017.00057)
Supplement: Supplementary file 1 [file DataSheet1.docx]

Supplementary Material

Persistent Exposure to *Porphyromonas gingivalis* Promotes Proliferative and Invasion Capabilities, and Tumorigenic Properties of Human Immortalized Oral Epithelial Cells

*Fengxue Geng, Junchao Liu, Yan Guo, Chen Li, Hongyang Wang, Hongyan Wang, Haijiao Zhao, Yaping Pan

*** Correspondence:** Yaping Pan [yppan@cmu.edu.cn](mailto:yppan@cmu.edu.cn)

**

**

**Figure 1** To confirm the appropriate infection condition induced by *P. gingivalis*, cells were repeatedly exposed to *P. gingivalis* every 3 or 4 days at an MOI of 10 or 1 (3 or 24 h/time). For example, cells from groups of 3 h were infected with *P. gingivalis* for 3 h and washed with PBS for 3 times; followed by another 21 h culture with fresh medium. Then, according to the cells condition after *P. gingivalis* challenging, cells in all the groups were subcultured or the medium was replaced. The proliferation rate of each infection group together with the non-infected control was determined 5 weeks later with the MTT method. As shown, the viability of HIOECs infected with *P. gingivalis* at an MOI of 10 decreased in both groups (24 or 3 h). The proliferation of cells infected with *P. gingivalis* at an MOI of 1 for 3 h (each time) was only significantly increased at the time point of 72 h (*P* < 0.0167). However, when HIOECs were infected with *P. gingivalis* at a lower MOI of 1 for 24 h each time, the proliferation rate was significantly increased comparing to the control (*P* < 0.0167) at all the time points.

B

A

C

D


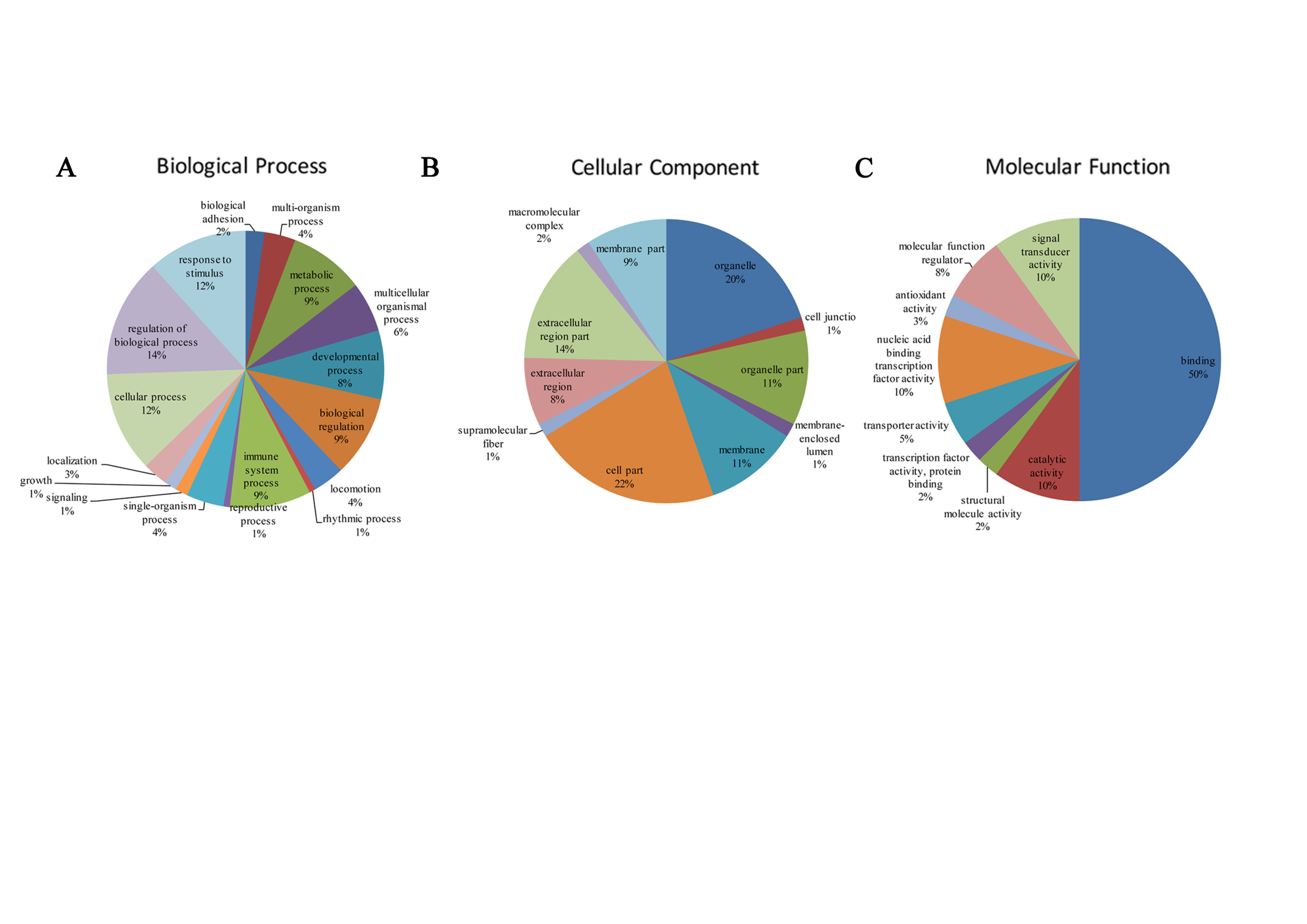


**Figure 4**

B

A

B

**Figure 6**

**Figure 2** GO analysis for biological process, cellular component and molecular function specific to selected genes was shown. A. The selected genes were involved in multiple biological processes such as regulation of biological process (14%), cellular process (12%), response to stimulus (12%) and immune system process (9%). B. The cellular component contained cell part (22), organelle (20%), extracellular region part (14%), membrane (11%), etc. C. The molecular function analysis (B) revealed that most of selected genes were of binding (50%), signal transducer activity (10%), catalytic activity (10%) and transcription factor activity (10%).

**Table 1**

| Gene symbol | *P* value | Fold change | Regulation | Clinical role | Cancer | Reference |
| --- | --- | --- | --- | --- | --- | --- |
| *CXCL10* | 0.029791 | 23.9495 | Up | a poor prognostic biomarker | colorectal cancer | (Bai et al., 2016) |
|  |  |  |  | a biomarker | poorly differentiated breast cancer | (Narita et al., 2016) |
|  |  |  |  | an independent predictor for poor survival | human pancreatic cancer | (Delitto et al., 2015) |
|  |  |  |  | a prognostic marker | advanced melanoma | (Jiang et al., 2015a) |
|  |  |  |  | a surrogate marker of host immunity | renal cell carcinoma | (Polimeno et al., 2013) |
| *CSF1* | 0.007512 | 23.04768 | Up | an indicated poor prognostic marker | lung adenocarcinoma | (Pei et al., 2015) |
|  |  |  |  | a predicted marker for progression and mortality | breast cancer | (Richardsen et al., 2015) |
|  |  |  |  | a predicted marker | lung cancer bone metastasis | (Hung et al., 2014) |
|  |  |  |  | a serum marker for poor overall survival | early breast cancer | (Aharinejad et al., 2013) |
|  |  |  |  | a marker correlated with shorter survival with no metastasis | non-small cell lung cancer (NSCLC). | (Skrzypski et al., 2013) |
|  |  |  |  | a poor prognostic marker | leiomyosarcoma. | (Espinosa et al., 2009) |
| *TNFAIP2* | 0.047035 | 14.66324 | Up | an independent prognostic indicator | nasopharyngeal carcinoma | (Chen et al., 2011) |
| *USP18* | 0.016806 | 11.08343 | Up | a prognostic marker (combined with DiGeorge syndrome critical region gene 2) | muscle invasive bladder cancer | (Kim et al., 2014) |
|  |  |  |  | an antineoplastic target | lung cancer | (Guo et al., 2012) |
| *NRCAM* | 2.73E-04 | 9.655933 | Up | an independent prognostic predictor | advanced colorectal cancer | (Chan et al., 2011) |
|  |  |  |  | implicated in the pathogenesis and behavior | papillary thyroid cancers | (Gorka et al., 2007) |
|  |  |  |  | a marker for detection | brain tumor | (Sehgal et al., 1998) |
| *NNMT* | 0.00424 | 7.07482 | Up | an independent prognosticator for patients' survival | pancreatic cancer | (Xu et al., 2016) |
|  |  |  |  | an early diagnostic biomarker | oral cancer | (Sartini et al., 2012) |
|  |  |  |  | a biomarker and a target for treatment | OSCC | (Emanuelli et al., 2010) |
|  |  |  |  | a serum marker for early detection | lung cancer | (Tomida et al., 2009) |
|  |  |  |  | a prognostic factor | hepatocellular carcinoma | (Kim et al., 2009) |
|  |  |  |  | a serum marker for early detection | colorectal cancer | (Roessler et al., 2005) |
|  |  |  |  | a potential biomarker | papillary thyroid carcinoma | (Xu et al., 2003) |
| *CYGB* | 0.004942 | 6.060973 | Up | an independent predictive factor of prognosis | Glioma | (Xu et al., 2013) |
| *CXCL11* | 0.044297 | 5.888966 | Up | an independent marker for prognosis | gastric adenocarcinoma | (Pasini et al., 2014) |
|  |  |  |  | a marker related to poor prognosis | colorectal cancer | (Zeng et al., 2016) |
|  |  |  |  | a diagnostic marker | triple negative breast carcinomas | (Narita et al., 2016) |
|  |  |  |  | a diagnostic marker combined with CXCL4 and CXCL9 | NSCLC | (Spaks et al., 2015) |
|  |  |  |  | a surrogate marker of host immunity | renal cell carcinoma | (Polimeno et al., 2013) |
| *FLI1* | 0.001262 | 5.813732 | Up | a diagnostic marker | phosphaturic mesenchymal tumors | (Tajima et al., 2015) |
|  |  |  |  | a prognostic factor | acute myeloid leukemia | (Kornblau et al., 2011) |
|  |  |  |  | a predicted marker | radiotherapy resistant oral squamous cell carcinoma | (Shintani et al., 2010) |
|  |  |  |  | a landmark | Ewing sarcoma | (Paronetto, 2013) |
| *WFDC2* | 1.30E-04 | 5.160519 | Up | a diagnostic marker | ovarian cancer | (Dikmen et al., 2015) |
|  |  |  |  | an independent determinant of poor prognosis | NSCLC | (Lamy et al., 2015) |
|  |  |  |  | a diagnostic biomarker | pancreatic adenocarcinoma | (Huang et al., 2015a) |
|  |  |  |  | a prognostic marker | endometrial cancer | (Capriglione et al., 2015) |
|  |  |  |  | a prognostic factor | gastric cancer | (Guo et al., 2015) |
| *SLPI* | 0.005033 | 5.159927 | Up | a salivary marker for high risk among ever  smokers | head and neck cancer | (Pierce Campbell et al., 2016) |
|  |  |  |  | a potential biomarker and a therapy target | castration-resistant prostate cancer. | (Zheng et al., 2016) |
|  |  |  |  | a salivary biomarker | OSCC | (Kawahara et al., 2016) |
|  |  |  |  | a prognostic biomarker for metastasis | OSCC | (Noorlag et al., 2015) |
|  |  |  |  | a diagnostic marker | epithelial ovarian cancer | (Carlson et al., 2013) |
|  |  |  |  | a prognostic marker | gastric cancer | (Cheng et al., 2008) |
| *LAMP3* | 0.005256 | 4.975458 | Up | a prognostic marker | esophageal squamous cell carcinoma. | (Liao et al., 2015) |
|  |  |  |  | an independent prognostic marker | gastrointestinal cancer | (Sun et al., 2014b) |
|  |  |  |  | a prognostic marker | head and neck cancer | (Nagelkerke et al., 2015) |
|  |  |  |  | an independent prognostic marker | breast cancer | (Nagelkerke et al., 2011) |
|  |  |  |  | a prognostic marker | cervical cancer | (Kanao et al., 2005) |
| *GRHL3* | 0.010426 | 4.635962 | Down | a prognostic and diagnostic factor | diffuse large B cell lymphoma | (Liu et al., 2016) |
| *SERPINA1* | 0.002098 | 4.352729 | Up | a salivary risk marker | OSCC | (Kawahara et al., 2016) |
|  |  |  |  | an early diagnostic biomarker | B-cell acute lymphoblastic leukemia | (Cavalcante Mde et al., 2016) |
|  |  |  |  | a predictor of survival | breast cancer | (Chan et al., 2015) |
|  |  |  |  | a prognostic biomarkers and candidate therapeutic targets | colorectal cancer | (Kwon et al., 2015) |
|  |  |  |  | a serum biomarker for detecting | gastric cancer | (Yang et al., 2015) |
|  |  |  |  | a candidate diagnostic biomarkers | prostate cancer | (Davalieva et al., 2015) |
|  |  |  |  | a tumor cell-associated biomarker for progression | cutaneous squamous cell carcinoma. | (Farshchian et al., 2011) |
|  |  |  |  | a potential marker | Insulinomas | (de Sa et al., 2007) |
| *GAS6* | 0.00145 | 3.492453 | Up | a serum marker for diagnosis and prognosis | OSCC | (Jiang et al., 2015b) |
|  |  |  |  | a factor associated with nodes involvement and tumour stage | colorectal cancer | (Martinelli et al., 2015) |
|  |  |  |  | an independent predictor of poor survival | ovarian cancer | (Buehler et al., 2013) |
| *CD274* | 0.044261 | 2.631684 | Up | an independent prognostic marker | OSCC | (Lin et al., 2015) |
|  |  |  |  | a biomarker of active tumor immunity | breast tumor | (Cimino-Mathews et al., 2016) |
|  |  |  |  | a prognostic marker | gastric cancer (Stage II/III) | (Tamura et al., 2015) |
|  |  |  |  | a prognostic marker | Bladder cancer | (Huang et al., 2015b) |
|  |  |  |  | an independent predictor of poor prognosis | non-small cell lung cancer | (Mao et al., 2015) |
| *SIX1* | 0.044448 | 2.462999 | Up | a predictor for outcome | prostate cancer. | (Zeng et al., 2015) |
|  |  |  |  | an independent prognostic marker | colorectal cancer | (Kahlert et al., 2015) |
|  |  |  |  | an independent prognostic biomarker | breast cancer | (Jin et al., 2014) |
|  |  |  |  | an independent prognostic biomarker | gastric adenocarcinoma | (Lv et al., 2014) |
| *TRIM29* | 0.043297 | 2.078059 | Down | a prognostic marker and a potent treatment target. | NSCLC | (Song et al., 2015) |
|  |  |  |  | a prognostic marker | pancreatic ductal adenocarcinoma | (Sun et al., 2014a) |
|  |  |  |  | a diagnostic marker from basal cells | prostate cancer | (Kanno et al., 2014) |
|  |  |  |  | a novel marker for cancer recurrence and survival | colorectal cancer | (Jiang et al., 2013) |
|  |  |  |  | an independent predictor for lymph node metastasis | gastric cancer | (Kosaka et al., 2007) |

The clinical roles of some differentially expressed genes from microarray data which have been considered as certain biomarkers for tumor detection, diagnosis, development or prognosis were concluded according to the recent clinical publications.

**Table 2**

|  | P value | Ratio | Regulation | Gene symbol | Description | Mainly subcellular location | Biological function |
| --- | --- | --- | --- | --- | --- | --- | --- |
| IPI00910385_MYADM | 0.0031 | 2.475954 | Up | MYADM | Myeloid-associated differentiation marker | cytoskeleton | [To positively regulate of cell migration](http://www.ebi.ac.uk/QuickGO/GTerm?id=GO:0030335) |
| IPI01009386_ZNF419 | 0.040398 | 2.329425 | Up | ZNF419 | Zinc Finger Protein 419 | Nucleus | Involved in transcriptional regulation, nucleic acid and protein binding |
| IPI00884099_AQPEP | 0.02232 | 2.081667 | Up | AQPEP | Aminopeptidase Q | Membrane | [Metalloaminopeptidase activity, signal transduction](http://www.ebi.ac.uk/QuickGO/GTerm?id=GO:0070006) |
| IPI00641957_SRSF11 | 0.038137 | 1.703736 | Up | SRSF11 | Serine/Arginine-Rich Splicing Factor 11 | Nucleus | May be involved in pre-mRNA processing and RNA splicing |
| IPI00020793_NIP7 | 0.021225 | 1.682463 | Up | NIP7 | Nucleolar Pre-RRNA Processing Protein | Nucleus | Involved in pre-rRNA processing and ribosome subunit assembly |
| IPI00328876_MYPOP | 0.039832 | 1.59149 | Up | MYPOP | Myb-related transcription factor, partner of profiling | Nucleus | Transcriptional repressor |
| IPI00014849_PDK3 | 0.025596 | 1.550787 | Up | PDK3 | Pyruvate Dehydrogenase Kinase, Isozyme 3 | mitochondrion | To participate in regulation of glucose metabolism and generation of reactive oxygen species. |
| IPI00178611_CCDC109B | 0.043684 | 0.496106 | down | CCDC109B | Coiled-Coil Domain-Containing Protein 109B | mitochondrion | To negatively regulate the mitochondrial inner membrane calcium uniporter activation and take part in cytoplasmic calcium signals and cell death pathways activation. |
| IPI00179757_KIF1C | 0.042375 | 0.477494 | down | KIF1C | Kinesin Family Member 1C | endoplasmic reticulum and golgi apparatus | RNA binding and microtubule binding. |
| IPI00748916_DOCK11 | 0.002893 | 0.436868 | down | DOCK11 | Dedicator Of Cytokinesis 11 | Cytosol | To activate CDC42 by exchanging bound GDP for free GTP. |

The mainly subcellular location and function of proteins above were based on Genecards (<http://www.genecards.org/> ) and Uniprot (<http://www.uniprot.org/>) database.

**REFERENCES**

Aharinejad, S., Salama, M., Paulus, P., Zins, K., Berger, A., and Singer, C.F. (2013). Elevated CSF1 serum concentration predicts poor overall survival in women with early breast cancer. *Endocr Relat Cancer* 20**,** 777-783.

Bai, M., Chen, X., and Ba, Y.I. (2016). CXCL10/CXCR3 overexpression as a biomarker of poor prognosis in patients with stage II colorectal cancer. *Mol Clin Oncol* 4**,** 23-30.

Buehler, M., Tse, B., Leboucq, A., Jacob, F., Caduff, R., Fink, D., Goldstein, D.R., and Heinzelmann-Schwarz, V. (2013). Meta-analysis of microarray data identifies GAS6 expression as an independent predictor of poor survival in ovarian cancer. *Biomed Res Int* 2013**,** 238284.

Capriglione, S., Plotti, F., Miranda, A., Ricciardi, R., Scaletta, G., Aloisi, A., Guzzo, F., Montera, R., and Angioli, R. (2015). Utility of tumor marker HE4 as prognostic factor in endometrial cancer: a single-center controlled study. *Tumour Biol* 36**,** 4151-4156.

Carlson, A.M., Maurer, M.J., Goergen, K.M., Kalli, K.R., Erskine, C.L., Behrens, M.D., Knutson, K.L., and Block, M.S. (2013). Utility of progranulin and serum leukocyte protease inhibitor as diagnostic and prognostic biomarkers in ovarian cancer. *Cancer Epidemiol Biomarkers Prev* 22**,** 1730-1735.

Cavalcante Mde, S., Torres-Romero, J.C., Lobo, M.D., Moreno, F.B., Bezerra, L.P., Lima, D.S., Matos, J.C., Moreira Rde, A., and Monteiro-Moreira, A.C. (2016). A panel of glycoproteins as candidate biomarkers for early diagnosis and treatment evaluation of B-cell acute lymphoblastic leukemia. *Biomark Res* 4**,** 1.

Chan, H.J., Li, H., Liu, Z., Yuan, Y.C., Mortimer, J., and Chen, S. (2015). SERPINA1 is a direct estrogen receptor target gene and a predictor of survival in breast cancer patients. *Oncotarget* 6**,** 25815-25827.

Chan, J.Y., Ong, C.W., and Salto-Tellez, M. (2011). Overexpression of neurone glial-related cell adhesion molecule is an independent predictor of poor prognosis in advanced colorectal cancer. *Cancer Sci* 102**,** 1855-1861.

Chen, L.C., Chen, C.C., Liang, Y., Tsang, N.M., Chang, Y.S., and Hsueh, C. (2011). A novel role for TNFAIP2: its correlation with invasion and metastasis in nasopharyngeal carcinoma. *Mod Pathol* 24**,** 175-184.

Cheng, W.L., Wang, C.S., Huang, Y.H., Liang, Y., Lin, P.Y., Hsueh, C., Wu, Y.C., Chen, W.J., Yu, C.J., Lin, S.R., and Lin, K.H. (2008). Overexpression of a secretory leukocyte protease inhibitor in human gastric cancer. *Int J Cancer* 123**,** 1787-1796.

Cimino-Mathews, A., Thompson, E., Taube, J.M., Ye, X., Lu, Y., Meeker, A., Xu, H., Sharma, R., Lecksell, K., Cornish, T.C., Cuka, N., Argani, P., and Emens, L.A. (2016). PD-L1 (B7-H1) expression and the immune tumor microenvironment in primary and metastatic breast carcinomas. *Hum Pathol* 47**,** 52-63.

Davalieva, K., Kiprijanovska, S., Komina, S., Petrusevska, G., Zografska, N.C., and Polenakovic, M. (2015). Proteomics analysis of urine reveals acute phase response proteins as candidate diagnostic biomarkers for prostate cancer. *Proteome Sci* 13**,** 2.

De Sa, S.V., Correa-Giannella, M.L., Machado, M.C., Krogh, K., De Almeida, M.Q., Albergaria Pereira, M.A., Coelho Siqueira, S.A., Patzina, R.A., Ibuki, F.S., Sogayar, M.C., Machado, M.C., and Giannella-Neto, D. (2007). Serpin peptidase inhibitor clade A member 1 as a potential marker for malignancy in insulinomas. *Clin Cancer Res* 13**,** 5322-5330.

Delitto, D., Perez, C., Han, S., Gonzalo, D.H., Pham, K., Knowlton, A.E., Graves, C.L., Behrns, K.E., Moldawer, L.L., Thomas, R.M., Liu, C., George, T.J., Jr., Trevino, J.G., Wallet, S.M., and Hughes, S.J. (2015). Downstream mediators of the intratumoral interferon response suppress antitumor immunity, induce gemcitabine resistance and associate with poor survival in human pancreatic cancer. *Cancer Immunol Immunother* 64**,** 1553-1563.

Dikmen, Z.G., Colak, A., Dogan, P., Tuncer, S., and Akbiyik, F. (2015). Diagnostic performances of CA125, HE4, and ROMA index in ovarian cancer. *Eur J Gynaecol Oncol* 36**,** 457-462.

Emanuelli, M., Santarelli, A., Sartini, D., Ciavarella, D., Rossi, V., Pozzi, V., Rubini, C., and Lo Muzio, L. (2010). Nicotinamide N-Methyltransferase upregulation correlates with tumour differentiation in oral squamous cell carcinoma. *Histol Histopathol* 25**,** 15-20.

Espinosa, I., Beck, A.H., Lee, C.H., Zhu, S., Montgomery, K.D., Marinelli, R.J., Ganjoo, K.N., Nielsen, T.O., Gilks, C.B., West, R.B., and Van De Rijn, M. (2009). Coordinate expression of colony-stimulating factor-1 and colony-stimulating factor-1-related proteins is associated with poor prognosis in gynecological and nongynecological leiomyosarcoma. *Am J Pathol* 174**,** 2347-2356.

Farshchian, M., Kivisaari, A., Ala-Aho, R., Riihila, P., Kallajoki, M., Grenman, R., Peltonen, J., Pihlajaniemi, T., Heljasvaara, R., and Kahari, V.M. (2011). Serpin peptidase inhibitor clade A member 1 (SerpinA1) is a novel biomarker for progression of cutaneous squamous cell carcinoma. *Am J Pathol* 179**,** 1110-1119.

Gorka, B., Skubis-Zegadlo, J., Mikula, M., Bardadin, K., Paliczka, E., and Czarnocka, B. (2007). NrCAM, a neuronal system cell-adhesion molecule, is induced in papillary thyroid carcinomas. *Br J Cancer* 97**,** 531-538.

Guo, Y., Chinyengetere, F., Dolinko, A.V., Lopez-Aguiar, A., Lu, Y., Galimberti, F., Ma, T., Feng, Q., Sekula, D., Freemantle, S.J., Andrew, A.S., Memoli, V., and Dmitrovsky, E. (2012). Evidence for the ubiquitin protease UBP43 as an antineoplastic target. *Mol Cancer Ther* 11**,** 1968-1977.

Guo, Y.D., Wang, J.H., Lu, H., Li, X.N., Song, W.W., Zhang, X.D., and Zhang, W.M. (2015). The human epididymis protein 4 acts as a prognostic factor and promotes progression of gastric cancer. *Tumour Biol* 36**,** 2457-2464.

Huang, T., Jiang, S.W., Qin, L., Senkowski, C., Lyle, C., Terry, K., Brower, S., Chen, H., Glasgow, W., Wei, Y., and Li, J. (2015a). Expression and diagnostic value of HE4 in pancreatic adenocarcinoma. *Int J Mol Sci* 16**,** 2956-2970.

Huang, Y., Zhang, S.D., Mccrudden, C., Chan, K.W., Lin, Y., and Kwok, H.F. (2015b). The prognostic significance of PD-L1 in bladder cancer. *Oncol Rep* 33**,** 3075-3084.

Hung, J.Y., Horn, D., Woodruff, K., Prihoda, T., Lesaux, C., Peters, J., Tio, F., and Abboud-Werner, S.L. (2014). Colony-stimulating factor 1 potentiates lung cancer bone metastasis. *Lab Invest* 94**,** 371-381.

Jiang, H., Gebhardt, C., Umansky, L., Beckhove, P., Schulze, T.J., Utikal, J., and Umansky, V. (2015a). Elevated chronic inflammatory factors and myeloid-derived suppressor cells indicate poor prognosis in advanced melanoma patients. *Int J Cancer* 136**,** 2352-2360.

Jiang, T., Liu, G., Wang, L., and Liu, H. (2015b). Elevated Serum Gas6 Is a Novel Prognostic Biomarker in Patients with Oral Squamous Cell Carcinoma. *PLoS One* 10**,** e0133940.

Jiang, T., Tang, H.M., Lu, S., Yan, D.W., Yang, Y.X., and Peng, Z.H. (2013). Up-regulation of tripartite motif-containing 29 promotes cancer cell proliferation and predicts poor survival in colorectal cancer. *Med Oncol* 30**,** 715.

Jin, H., Cui, M., Kong, J., Cui, X., Lin, Z., Wu, Q., and Liu, S. (2014). Sineoculis homeobox homolog 1 protein is associated with breast cancer progression and survival outcome. *Exp Mol Pathol* 97**,** 247-252.

Kahlert, C., Lerbs, T., Pecqueux, M., Herpel, E., Hoffmeister, M., Jansen, L., Brenner, H., Chang-Claude, J., Blaker, H., Kloor, M., Roth, W., Pilarsky, C., Rahbari, N.N., Scholch, S., Bork, U., Reissfelder, C., Weitz, J., Aust, D., and Koch, M. (2015). Overexpression of SIX1 is an independent prognostic marker in stage I-III colorectal cancer. *Int J Cancer* 137**,** 2104-2113.

Kanao, H., Enomoto, T., Kimura, T., Fujita, M., Nakashima, R., Ueda, Y., Ueno, Y., Miyatake, T., Yoshizaki, T., Buzard, G.S., Tanigami, A., Yoshino, K., and Murata, Y. (2005). Overexpression of LAMP3/TSC403/DC-LAMP promotes metastasis in uterine cervical cancer. *Cancer Res* 65**,** 8640-8645.

Kanno, Y., Watanabe, M., Kimura, T., Nonomura, K., Tanaka, S., and Hatakeyama, S. (2014). TRIM29 as a novel prostate basal cell marker for diagnosis of prostate cancer. *Acta Histochem* 116**,** 708-712.

Kawahara, R., Bollinger, J.G., Rivera, C., Ribeiro, A.C., Brandao, T.B., Paes Leme, A.F., and Maccoss, M.J. (2016). A targeted proteomic strategy for the measurement of oral cancer candidate biomarkers in human saliva. *Proteomics* 16**,** 159-173.

Kim, J., Hong, S.J., Lim, E.K., Yu, Y.S., Kim, S.W., Roh, J.H., Do, I.G., Joh, J.W., and Kim, D.S. (2009). Expression of nicotinamide N-methyltransferase in hepatocellular carcinoma is associated with poor prognosis. *J Exp Clin Cancer Res* 28**,** 20.

Kim, Y.H., Kim, W.T., Jeong, P., Ha, Y.S., Kang, H.W., Yun, S.J., Moon, S.K., Choi, Y.H., Kim, I.Y., and Kim, W.J. (2014). Novel combination markers for predicting survival in patients with muscle invasive bladder cancer: USP18 and DGCR2. *J Korean Med Sci* 29**,** 351-356.

Kornblau, S.M., Qiu, Y.H., Zhang, N., Singh, N., Faderl, S., Ferrajoli, A., York, H., Qutub, A.A., Coombes, K.R., and Watson, D.K. (2011). Abnormal expression of FLI1 protein is an adverse prognostic factor in acute myeloid leukemia. *Blood* 118**,** 5604-5612.

Kosaka, Y., Inoue, H., Ohmachi, T., Yokoe, T., Matsumoto, T., Mimori, K., Tanaka, F., Watanabe, M., and Mori, M. (2007). Tripartite motif-containing 29 (TRIM29) is a novel marker for lymph node metastasis in gastric cancer. *Ann Surg Oncol* 14**,** 2543-2549.

Kwon, C.H., Park, H.J., Choi, J.H., Lee, J.R., Kim, H.K., Jo, H.J., Kim, H.S., Oh, N., Song, G.A., and Park Do, Y. (2015). Snail and serpinA1 promote tumor progression and predict prognosis in colorectal cancer. *Oncotarget* 6**,** 20312-20326.

Lamy, P.J., Plassot, C., and Pujol, J.L. (2015). Serum HE4: An Independent Prognostic Factor in Non-Small Cell Lung Cancer. *PLoS One* 10**,** e0128836.

Liao, X., Chen, Y., Liu, D., Li, F., Li, X., and Jia, W. (2015). High Expression of LAMP3 Is a Novel Biomarker of Poor Prognosis in Patients with Esophageal Squamous Cell Carcinoma. *Int J Mol Sci* 16**,** 17655-17667.

Lin, Y.M., Sung, W.W., Hsieh, M.J., Tsai, S.C., Lai, H.W., Yang, S.M., Shen, K.H., Chen, M.K., Lee, H., Yeh, K.T., and Chen, C.J. (2015). High PD-L1 Expression Correlates with Metastasis and Poor Prognosis in Oral Squamous Cell Carcinoma. *PLoS One* 10**,** e0142656.

Liu, W., Ha, M., Wang, X., and Yin, N. (2016). Clinical significance of GRHL3 expression in diffuse large B cell lymphoma. *Tumour Biol*.

Lv, H., Cui, A., Sun, F., Zhang, Y., Li, Y., Li, L., and Lin, Z. (2014). Sineoculis homeobox homolog 1 protein as an independent biomarker for gastric adenocarcinoma. *Exp Mol Pathol* 97**,** 74-80.

Mao, Y., Li, W., Chen, K., Xie, Y., Liu, Q., Yao, M., Duan, W., Zhou, X., Liang, R., and Tao, M. (2015). B7-H1 and B7-H3 are independent predictors of poor prognosis in patients with non-small cell lung cancer. *Oncotarget* 6**,** 3452-3461.

Martinelli, E., Martini, G., Cardone, C., Troiani, T., Liguori, G., Vitagliano, D., Napolitano, S., Morgillo, F., Rinaldi, B., Melillo, R.M., Liotti, F., Nappi, A., Bianco, R., Berrino, L., Ciuffreda, L.P., Ciardiello, D., Iaffaioli, V., Botti, G., Ferraiolo, F., and Ciardiello, F. (2015). AXL is an oncotarget in human colorectal cancer. *Oncotarget* 6**,** 23281-23296.

Nagelkerke, A., Mujcic, H., Bussink, J., Wouters, B.G., Van Laarhoven, H.W., Sweep, F.C., and Span, P.N. (2011). Hypoxic regulation and prognostic value of LAMP3 expression in breast cancer. *Cancer* 117**,** 3670-3681.

Nagelkerke, A., Sweep, F.C., Stegeman, H., Grenman, R., Kaanders, J.H., Bussink, J., and Span, P.N. (2015). Hypoxic regulation of the PERK/ATF4/LAMP3-arm of the unfolded protein response in head and neck squamous cell carcinoma. *Head Neck* 37**,** 896-905.

Narita, D., Seclaman, E., Anghel, A., Ilina, R., Cireap, N., Negru, S., Sirbu, I.O., Ursoniu, S., and Marian, C. (2016). Altered levels of plasma chemokines in breast cancer and their association with clinical and pathological characteristics. *Neoplasma* 63**,** 141-149.

Noorlag, R., Van Der Groep, P., Leusink, F.K., Van Hooff, S.R., Frank, M.H., Willems, S.M., and Van Es, R.J. (2015). Nodal metastasis and survival in oral cancer: Association with protein expression of SLPI, not with LCN2, TACSTD2, or THBS2. *Head Neck* 37**,** 1130-1136.

Paronetto, M.P. (2013). Ewing sarcoma protein: a key player in human cancer. *Int J Cell Biol* 2013**,** 642853.

Pasini, F.S., Zilberstein, B., Snitcovsky, I., Roela, R.A., Mangone, F.R., Ribeiro, U., Jr., Nonogaki, S., Brito, G.C., Callegari, G.D., Cecconello, I., Alves, V.A., Eluf-Neto, J., Chammas, R., and Federico, M.H. (2014). A gene expression profile related to immune dampening in the tumor microenvironment is associated with poor prognosis in gastric adenocarcinoma. *J Gastroenterol* 49**,** 1453-1466.

Pei, B., Sun, B., Zhang, Y., Wang, A., and Zhang, Z. (2015). [Expression of colony-stimulating factor 1 in lung adenocarcinoma and its prognostic implication]. *Zhonghua Zhong Liu Za Zhi* 37**,** 113-118.

Pierce Campbell, C.M., Giuliano, A.R., Torres, B.N., O'keefe, M.T., Ingles, D.J., Anderson, R.L., Teras, L.R., and Gapstur, S.M. (2016). Salivary secretory leukocyte protease inhibitor (SLPI) and head and neck cancer: The Cancer Prevention Study II Nutrition Cohort. *Oral Oncol* 55**,** 1-5.

Polimeno, M., Napolitano, M., Costantini, S., Portella, L., Esposito, A., Capone, F., Guerriero, E., Trotta, A., Zanotta, S., Pucci, L., Longo, N., Perdona, S., Pignata, S., Castello, G., and Scala, S. (2013). Regulatory T cells, interleukin (IL)-6, IL-8, vascular endothelial growth factor (VEGF), CXCL10, CXCL11, epidermal growth factor (EGF) and hepatocyte growth factor (HGF) as surrogate markers of host immunity in patients with renal cell carcinoma. *BJU Int* 112**,** 686-696.

Richardsen, E., Uglehus, R.D., Johnsen, S.H., and Busund, L.T. (2015). Macrophage-colony stimulating factor (CSF1) predicts breast cancer progression and mortality. *Anticancer Res* 35**,** 865-874.

Roessler, M., Rollinger, W., Palme, S., Hagmann, M.L., Berndt, P., Engel, A.M., Schneidinger, B., Pfeffer, M., Andres, H., Karl, J., Bodenmuller, H., Ruschoff, J., Henkel, T., Rohr, G., Rossol, S., Rosch, W., Langen, H., Zolg, W., and Tacke, M. (2005). Identification of nicotinamide N-methyltransferase as a novel serum tumor marker for colorectal cancer. *Clin Cancer Res* 11**,** 6550-6557.

Sartini, D., Pozzi, V., Renzi, E., Morganti, S., Rocchetti, R., Rubini, C., Santarelli, A., Lo Muzio, L., and Emanuelli, M. (2012). Analysis of tissue and salivary nicotinamide N-methyltransferase in oral squamous cell carcinoma: basis for the development of a noninvasive diagnostic test for early-stage disease. *Biol Chem* 393**,** 505-511.

Sehgal, A., Boynton, A.L., Young, R.F., Vermeulen, S.S., Yonemura, K.S., Kohler, E.P., Aldape, H.C., Simrell, C.R., and Murphy, G.P. (1998). Cell adhesion molecule Nr-CAM is over-expressed in human brain tumors. *Int J Cancer* 76**,** 451-458.

Shintani, S., Hamakawa, H., Nakashiro, K., Shirota, T., Hatori, M., Tanaka, M., Kuroshita, Y., and Kurokawa, Y. (2010). Friend leukaemia insertion (Fli)-1 is a prediction marker candidate for radiotherapy resistant oral squamous cell carcinoma. *Int J Oral Maxillofac Surg* 39**,** 1115-1119.

Skrzypski, M., Dziadziuszko, R., Jassem, E., Szymanowska-Narloch, A., Gulida, G., Rzepko, R., Biernat, W., Taron, M., Jelitto-Gorska, M., Marjanski, T., Rzyman, W., Rosell, R., and Jassem, J. (2013). Main histologic types of non-small-cell lung cancer differ in expression of prognosis-related genes. *Clin Lung Cancer* 14**,** 666-673 e662.

Song, X., Fu, C., Yang, X., Sun, D., Zhang, X., and Zhang, J. (2015). Tripartite motif-containing 29 as a novel biomarker in non-small cell lung cancer. *Oncol Lett* 10**,** 2283-2288.

Spaks, A., Jaunalksne, I., Spaka, I., Chudasama, D., Pirtnieks, A., and Krievins, D. (2015). Diagnostic Value of Circulating CXC Chemokines in Non-small Cell Lung Cancer. *Anticancer Res* 35**,** 6979-6983.

Sun, H., Dai, X., and Han, B. (2014a). TRIM29 as a novel biomarker in pancreatic adenocarcinoma. *Dis Markers* 2014**,** 317817.

Sun, R., Wang, X., Zhu, H., Mei, H., Wang, W., Zhang, S., and Huang, J. (2014b). Prognostic value of LAMP3 and TP53 overexpression in benign and malignant gastrointestinal tissues. *Oncotarget* 5**,** 12398-12409.

Tajima, S., Takashi, Y., Ito, N., Fukumoto, S., and Fukuyama, M. (2015). ERG and FLI1 are useful immunohistochemical markers in phosphaturic mesenchymal tumors. *Med Mol Morphol*.

Tamura, T., Ohira, M., Tanaka, H., Muguruma, K., Toyokawa, T., Kubo, N., Sakurai, K., Amano, R., Kimura, K., Shibutani, M., Maeda, K., and Hirakawa, K. (2015). Programmed Death-1 Ligand-1 (PDL1) Expression Is Associated with the Prognosis of Patients with Stage II/III Gastric Cancer. *Anticancer Res* 35**,** 5369-5376.

Tomida, M., Mikami, I., Takeuchi, S., Nishimura, H., and Akiyama, H. (2009). Serum levels of nicotinamide N-methyltransferase in patients with lung cancer. *J Cancer Res Clin Oncol* 135**,** 1223-1229.

Xu, H.W., Huang, Y.J., Xie, Z.Y., Lin, L., Guo, Y.C., Zhuang, Z.R., Lin, X.P., Zhou, W., Li, M., Huang, H.H., Wei, X.L., Man, K., and Zhang, G.J. (2013). The expression of cytoglobin as a prognostic factor in gliomas: a retrospective analysis of 88 patients. *BMC Cancer* 13**,** 247.

Xu, J., Moatamed, F., Caldwell, J.S., Walker, J.R., Kraiem, Z., Taki, K., Brent, G.A., and Hershman, J.M. (2003). Enhanced expression of nicotinamide N-methyltransferase in human papillary thyroid carcinoma cells. *J Clin Endocrinol Metab* 88**,** 4990-4996.

Xu, Y., Liu, P., Zheng, D.H., Wu, N., Zhu, L., Xing, C., and Zhu, J. (2016). Expression profile and prognostic value of NNMT in patients with pancreatic cancer. *Oncotarget*.

Yang, J., Xiong, X., Wang, X., Guo, B., He, K., and Huang, C. (2015). Identification of peptide regions of SERPINA1 and ENOSF1 and their protein expression as potential serum biomarkers for gastric cancer. *Tumour Biol* 36**,** 5109-5118.

Zeng, J., Shi, R., Cai, C.X., Liu, X.R., Song, Y.B., Wei, M., and Ma, W.L. (2015). Increased expression of Six1 correlates with progression and prognosis of prostate cancer. *Cancer Cell Int* 15**,** 63.

Zeng, Y.J., Lai, W., Wu, H., Liu, L., Xu, H.Y., Wang, J., and Chu, Z.H. (2016). Neuroendocrine-like cells -derived CXCL10 and CXCL11 induce the infiltration of tumor-associated macrophage leading to the poor prognosis of colorectal cancer. *Oncotarget*.

Zheng, D., Gui, B., Gray, K.P., Tinay, I., Rafiei, S., Huang, Q., Sweeney, C.J., Kibel, A.S., and Jia, L. (2016). Secretory leukocyte protease inhibitor is a survival and proliferation factor for castration-resistant prostate cancer. *Oncogene*.

**
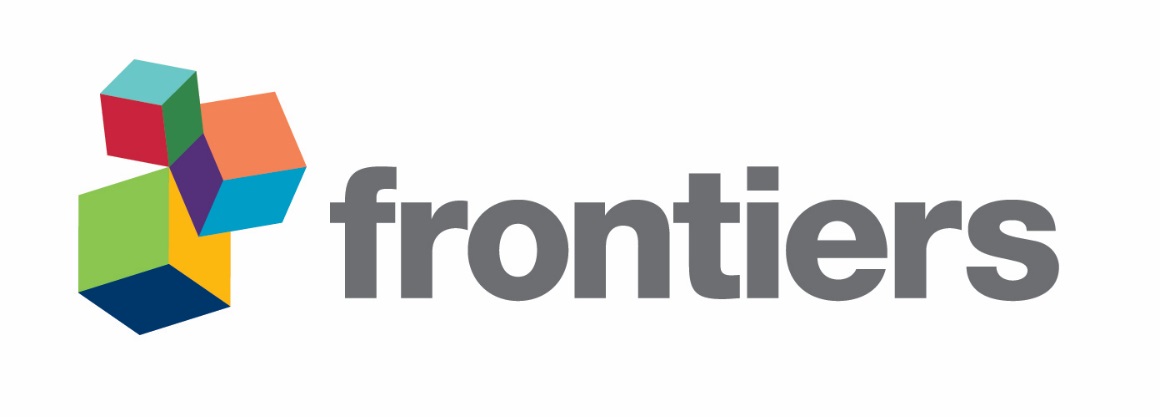
**
